# A case study: temporal trends of environmental stressors and reproductive health of smallmouth bass (*Micropterus dolomieu*) from a site in the Potomac River Watershed, Maryland, USA

**DOI:** 10.1007/s10646-022-02605-8

**Published:** 2022-12-01

**Authors:** Heather L. Walsh, Stephanie E. Gordon, Adam J. Sperry, Michael Kashiwagi, John Mullican, Vicki S. Blazer

**Affiliations:** 1 U.S. Geological Survey, Eastern Ecological Science Center, Leetown Research Laboratory, 11649 Leetown Rd., Kearneysville, WV 25430 USA; 2 Maryland Department of Natural Resources, Fishing and Boating Services, 10932 Putman Rd., Thurmont, MD 21788 USA; 3 Maryland Department of Natural Resources, Fishing and Boating Services, 20901 Fish Hatchery Rd., Hagerstown, MD 21740 USA

**Keywords:** Reproductive endocrine disruption, Testicular oocytes, Plasma vitellogenin, Contaminants, Land use, Long-term monitoring

## Abstract

Decades of poor reproductive success and young-of-the-year survival, combined with adult mortality events, have led to a decline in the smallmouth bass (SMB; *Micropterus dolomieu*) population in sections of the Potomac River. Previous studies have identified numerous biologic and environmental stressors associated with negative effects on SMB health. To better understand the impact of these stressors, this study was conducted at the confluence of Antietam Creek and the Potomac River from 2013 to 2019 to identify temporal changes associated with SMB reproductive health. Surface water samples were collected and analyzed for over 300 organic contaminants, including pesticides, phytoestrogens, pharmaceuticals, hormones and total estrogenicity (E2Eq). Adult SMB were collected and sampled for multiple endpoints, including gene transcripts associated with reproduction (molecular), histopathology (cellular), and organosomatic indices (tissue). In males, biomarkers of estrogenic endocrine disruption, including testicular oocytes (TO) and plasma vitellogenin (Vtg) were assessed. Numerous agriculture-related contaminants or land use patterns were associated with gene transcript abundance in both male and female SMB. Positive associations between pesticides in the immediate catchment with TO severity and E2Eq with plasma Vtg in males were identified. In males, the prevalence of TO and detectable levels of plasma Vtg, liver vitellogenin transcripts (*vtg*) and testis *vtg* were high throughout the study. Peaks of complex mixtures of numerous contaminants occurred during the spring/early summer when spawning and early development occurs and to a lesser extent in fall/winter during recrudescence. Management practices to reduce exposure during these critical and sensitive periods may enhance reproductive health of these economically important sportfishes.

## Introduction

Smallmouth bass *Micropterus dolomieu* (SMB) are an economically important sportfish managed by state agencies within the Chesapeake Bay drainage. Over the past four decades, population declines in subwatersheds of the Chesapeake Bay have led to management concerns, particularly associated with SMB health. Co-infections of opportunistic bacterial pathogens, including *Aeromonas* spp. and *Flavobacterium columnare*, myxozoan, trematode and cestode parasites, and largemouth bass virus (LMBV) have been identified; however, no single factor is directly or consistently associated with mortality (Blazer et al. 2010). During investigations into adult fish kills throughout the Potomac River drainage, histopathological examination identified evidence of reproductive endocrine disruption including a high prevalence of intersex or testicular oocytes (TO) and elevated plasma vitellogenin (Vtg) concentrations in male bass (Blazer et al. 2007; Iwanowicz et al. 2009; Blazer et al. 2012). Similar findings of estrogenic endocrine disruption were also documented in the Susquehanna River drainage (Blazer et al. 2014a).

In multiple fish species, estrogenic endocrine disruption biomarkers such as TO and plasma Vtg have been associated with a variety of chemical exposures including natural and synthetic hormones (Rempel and Schlenk 2008; Sumpter and Jobling 2013), pesticides/herbicides (Bizarro et al. 2014; Kolpin et al. 2013), polycyclic aromatic hydrocarbons (PAHs; Grieshaber et. al 2018), polychlorinated biphenyls (PCBs; Baldigo et al. 2006; Lee Pow et al. 2017), pharmaceuticals (Niemuth et al. 2015; Palace et al. 2009), and wastewater effluent (Bahamonde et al. 2015; Tetreault et al. 2011; Woodling et al. 2006). In environmental monitoring studies, SMB are increasingly recognized as an indicator species sensitive to endocrine disruption (Hinck et al. 2009; Blazer et al. 2012, 2018; Iwanowicz et al. 2016; Abdel-moneim et al. 2017; Kadlec et al. 2017; Grieshaber et al. 2018). Initial studies within the Potomac drainage addressed site comparisons and potential contributing factors of endocrine disruption in discrete water or sediment samples collected at the time of adult SMB sampling. Positive correlations of TO prevalence and severity with atrazine in the water column and with total hormone/sterol in the bed sediment around spawning nests were noted in SMB collected during the spring in the Potomac drainage (Blazer et al. 2012; Kolpin et al. 2013). At sites within the Susquehanna drainage surface water estrone concentrations were correlated with TO severity and plasma Vtg in male SMB collected in the summer (Blazer et al. 2014a). Several studies nationwide have found associations between these endpoints and agricultural land use (Abdel-moneim et al. 2015; Blazer et al. 2012, 2014a) as well as industrial endocrine active chemicals (Grieshaber et al. 2018). A retrospective analysis of numerous studies on estrogenic endocrine disruption throughout the Chesapeake Bay watershed, using correlation and regression tree models, illustrated the importance of scale (immediate versus upstream catchment) and the landscape groups associated with endocrine disruption indicators measured (i.e., plasma Vtg and TO). It was shown that the more disturbed landscapes, such as agricultural land cover attributes (i.e. percent cultivated, pesticide application, phytoestrogen cover crops, as well as developed land cover attributes such as population density, road density and impervious surfaces) had positive relationships with estrogenic indicators, while percent forest and shrubs generally had a negative association (Blazer et al. 2021a).

Ultimately management agencies are concerned with maintaining healthy populations and studies in a variety of fish species suggest reproductive endocrine disruption may contribute to reduced populations (Jobling et al 2002) or even population collapses (Kidd et al. 2007). In SMB, males with TO were found to have reduced sperm motility and abundance (Blazer et al. 2012). Aside from land-use, other environmental variables can contribute to population effects including climatic factors and infectious disease, alone or in combination with contaminants. High flows during egg incubation or early fry/larval development have been linked to decreased juvenile abundance due to nest failures (Lukas and Orth 1995; Smith et al. 2005). Based on long term (1975–2017) Maryland Department of Natural Resources (MD DNR) juvenile abundance (catch per unit effort; CPUE) data, the overall abundance of SMB in regions of the Potomac River has decreased over the past four decades (Hitt et al. 2020). It was hypothesized that this decrease coincides with increasing spring flow variability and life history traits of SMB. It was further suggested that changes in fish abundance could not be explained by changes in water quality (Hitt et al. 2020). However, water quality was narrowly defined as nutrient and sediment loads and did not consider increased pesticide and other contaminant concentrations that can occur during runoff events (Phillips and Bode 2004; Lefrancq et al. 2017; Chen et al. 2019) or that while total pesticide mass has declined in the Chesapeake Bay watershed, toxicity units (or potency) have remained similar or increased (Hartwell 2011). In a modeling framework for ecological risk assessment developed by Li et al. (2020) to assess population-level effects of temperature, flow, and chemical exposure, warm summer water temperatures and year-round high flows had the most severe impacts on SMB populations. An increase in exposure to estrogenic endocrine disruptors both year-round and in early summer substantially reduced population size. However, acute exposures during the spawning season were more detrimental to the population than chronic exposures.

The above studies suggest multiple stressors are influencing SMB health and abundance and timing of stressor exposure may be important. Water and/or sediment samples taken at the time of adult fish sampling for biological effects may not be indicative of contaminant exposure during sensitive developmental periods. In fish, the periods of sexual differentiation in juveniles (Duffy et al. 2014; Kiparissis et al. 2003; Koger et al. 2000; Liao et al. 2014) and recrudescence in adults (Ankley and Johnson 2004) are important life stages sensitive to endocrine disruption. Consequently, temporal sampling over multiple years is necessary to assess both climatic factors such as variability in flow and variations in chemical contaminant exposure. Hence, the goals of this report were to (1) document the temporal (seasonal and annual) variation in chemical concentrations during key developmental periods; (2) to evaluate the utility of monthly/bimonthly sampling of surface water to identify exposure to multiple stressors that may impact SMB and other economically important fishes and (3) identify associations among indicators of reproductive endocrine disruption, gene transcript abundance in liver and testes, changes in contaminant exposure, and other environmental variables and land-use attributes.

## Methods

### Land use analyses

**Fig. 1:**
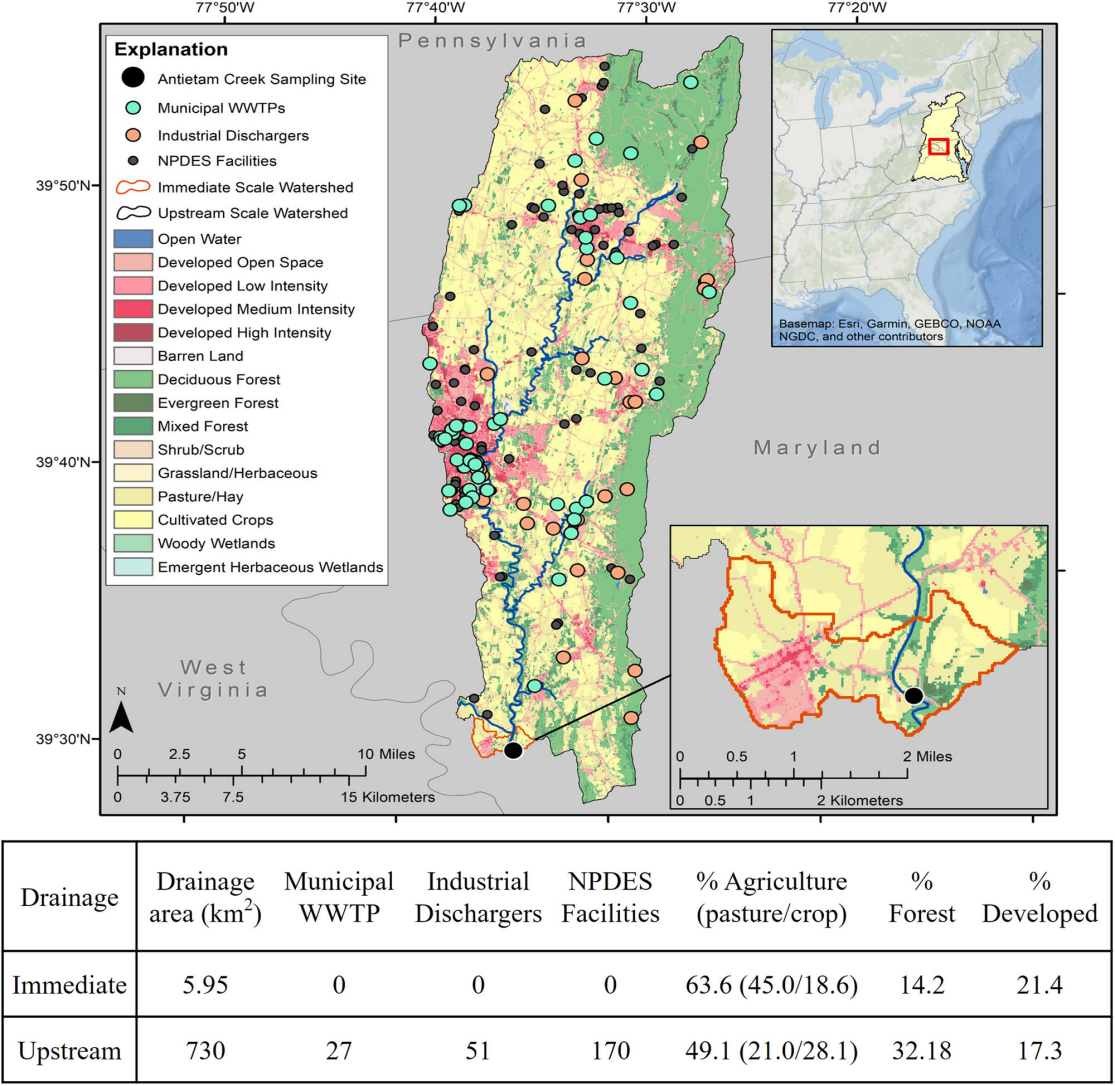
Land use (2016) in the immediate and upstream catchments around the smallmouth bass, Antietam Creek – Potomac Mainstem collection site near Dargan, Maryland

The location of the Antietam Creek – Potomac Mainstem sampling site (Fig. 1) near the confluence of Antietam Creek and the Potomac River, near Dargan, Maryland (39.44978, −77.73019) was used to identify the immediate (local) NHDPlus catchment (NHDPlus Version 2.1; EPA and USGS, 2012) in ArcMap 10.6 (Esri, Redlands, California) using a spatial join.

The upstream catchment was manually delineated by selecting upstream NHDPlus catchments to reflect the watershed area using the nearby USGS Streamgage (USGS 01619500). Landcover for 2016 was downloaded from the National Landcover Database (https://www.mrlc.gov/data, Homer et al. 2020) and was summarized at both scales using the zonal histogram tool and calculating percent coverage as ((#of cells per class per area) / (total # of cells per area) × 100). The presence of wastewater treatment plants (WWTP) and National Pollutant Discharge Elimination System (NPDES) facilities (see Blazer et al. 2021b for details), and estimated total pesticide application, percent high-phytoestrogen crop cover, and total nutrients from applied biosolids were calculated for the immediate and upstream catchment for all years (Fig. 1). High-phytoestrogen crop cover was calculated as percent of land from the National Agricultural Statistics Service Cropland Data Layer (USDA, 2021) with crops identified as having high levels of phytoestrogens (alfalfa, barley, clover/wildflowers, flaxseed, grapes, oats, peanuts, barley, rye, soybeans, hops and wheat) present either all year or in crop rotation using previous methods (Gordon et al. 2017). Data on biosolids was available from the Chesapeake Scenario Assessment Tool (https://cast.chesapeakebay.net/) as estimated nutrient (nitrogen and phosphorus) applications from biosolids at the county scale for all counties intersecting the Chesapeake Bay Watershed during a water year (October 1 to September 31). The application rate (pounds/acre) was applied to acres of pasture, turf grass landcovers (tree canopy over turf grass, fractional turf grass (large)) and cropland/pasture per catchment as defined in the 1 m High Resolution Chesapeake Landcover (Chesapeake Conservancy; https://www.chesapeakeconservancy.org/conservation-innovation-center/high-resolution-data/land-cover-data-project/). These estimates were summed into total pounds of nutrients applied from biosolids applications and converted to kilograms. Summaries for pesticide applications were generated using state-level annual agricultural pesticide-use estimates by crop or crop group (Wieben 2021), originally provided by the US Department of Agriculture, which were apportioned to appropriate crop types identified in the USDA National Cropland Data Layer (available from USDA National Agricultural Statistics Service https://www.nass.usda.gov/Research_and_Science/Cropland/Release/index.php) (Table 1).

**Table 1:** Total estimated pesticide application, % high phytoestrogen crop cover, and total nutrients from applied biosolids in the immediate and upstream catchments of the smallmouth bass sampling site in Antietam, Maryland from 2013 to 2019

Catchment	Total pesticide application (kg/year)	% High-phytoestrogen crop cover	Total nutrients from applied biosolids (kg)
Immediate 2013	1149.27	7.61	0.14
Immediate 2014	981.41	3.60	0.26
Immediate 2015	651.46	14.32	0.25
Immediate 2016	820.24	14.24	0.38
Immediate 2017	827.03	13.09	0.18
Immediate 2018	739.51	17.79	0.25
Immediate 2019	614.34	9.51	49.42
Upstream 2013	211,882.23	4.49	41,826.11
Upstream 2014	203,845.10	4.83	40,719.85
Upstream 2015	253,055.29	6.72	40,521.92
Upstream 2016	196,743.87	6.77	40,326.83
Upstream 2017	197,642.21	6.17	40,194.05
Upstream 2018	204,996.47	6.41	40,213.21
Upstream 2019	158,244.42	5.92	43,170.63

### Surface water analyses

Water samples were collected bimonthly during the spring (April–June) and once a month the remainder of the year (July–March) when possible, over the course of this study. Attempts were also made to capture at least two high flow events per year. Sample collection and analyses have been previously described (Smalling et al. 2021). Surface water samples were analyzed for approximately 300 organic contaminants including 105 current-use pesticides (Hladik et al. 2008) at the U.S. Geological Survey (USGS) Organic Chemistry Research Laboratory (CA), Sacramento, California, 107 pharmaceuticals (Furlong et al. 2014), 20 hormones and sterols (Foreman et al. 2012) and 52 wastewater indicators (Zaugg et al. 2007) at the USGS National Water Quality Laboratory (NWQL), Denver, Colorado, and 7 phytoestrogens and 8 mycotoxins (Yost et al. 2013) at the USGS Organic Geochemistry Research Laboratory, Lawrence, Kansas. Not all chemical schedules were conducted at all time periods. Details on methods, reporting limits for each compound and the complete data set can be found in Williams et al. (2019). In 2018 through 2019 an alternate pesticide method (NWQL) was used which quantified 108 parent compounds and 116 transformation products (Sandstrom et al. 2015).

Total estrogenicity (E2Eq) was quantified from water samples with a bioluminescent yeast-based estrogen screen (BLYES) and reported relative to 17β-estradiol equivalents as described in Ciparis et al. (2012). The assay indicates the presence of compounds that can bind to the estrogen receptors but does not differentiate between agonists and antagonists (Sanseverino et al. 2005).

### Smallmouth bass sampling

Approximately 20 adult SMB (≥200 mm in length) were collected for each sampling effort by boat electroshocking in spring of 2013, 2015–2019 and in the fall of 2016 and 2018. All fish were humanely euthanized with a lethal dose (350 mg/L) of tricaine methanesulfonate (MS-222, Argent Finquel, Redmond, Washington) according to the USGS Eastern Ecological Science Center’s Institutional Animal Care and Use Committee protocol. Fish were weighed (gm), measured (total length in mm), examined for visible abnormalities and a blood sample was obtained from the caudal vessel using a sterile 3 mL syringe with a 23-gauge needle. Blood was placed into a heparinized Vacutainer tube (Thermo Fisher Scientific, Waltham, Massachusetts, USA) and stored on wet ice until returned to the laboratory (2–4 h). Blood was centrifuged at 1000 × g at 4 °C for 10 min and plasma was aliquoted into cryovials and stored at −80 °C. A ventral slit was cut from the vent to the operculum and the gonad and liver were excised and weighed to the nearest 0.01 g. Pieces of gonad tissue were placed into bottles containing Z-Fix® (Anatech Ltd, Battle Creek, Michigan) for histology and small pieces of liver and testes were placed into separate RNALater® (Thermo Fisher Scientific) tubes for RNA preservation. Samples preserved in RNALater® were kept in the refrigerator for 24 hr prior to freezing at −20 °C until further use. The gonadosomatic index (GSI) was calculated as follows: (gonad weight / body weight) × 100.

### Laboratory analyses

Pieces of gonad were fixed in Z-Fix® (Anatech, Battle Creek, Michigan) for ≥48 h prior to being processed for histology. Tissue sections were embedded in paraffin, sectioned at 5 µm, and stained with hematoxylin and eosin (Luna 1992). Five cross-sections along the length of one testis were examined for the presence of TO and a severity rating was assigned according to Blazer et al. (2007) by taking the mean TO severity assigned to each of the five sections. Testicular oocytes were never found to advance past the primordial follicle stage. A severity score of 1 indicated a single oocyte per field (4× objective or 24 mm^2^), 2 was two or more oocytes per field not closely associated, 3 was multiple clusters of 2–5 oocytes closely associated, and 4 was zonal groups of more than five closely associated oocytes. A ranking system was also established to classify TO severity as low, medium, or high. Low ranged from mean TO severity scores of 0–0.4, medium ranged from 0.5 to 1.5, and high was >1.6.

Plasma Vtg concentrations were measured using a direct enzyme-linked immunosorbent assay (ELISA) with monoclonal antibody 3G2 (Caymen Chemical, Ann Arbor, Michigan) as previously described (Denslow et al. 1999; Blazer et al. 2014b). Briefly, plasma samples were diluted as necessary in PBSZ-AP (10 mM phosphate, 150 mM NaCl, 0.02% azide, pH 7.6). SMB plasma Vtg was used as a standard for all plasma analyzed. The plasma Vtg standards were prepared at the University of Florida, Department of Physiological Sciences from plasma of 17β-estradiol exposed male SMB held at the USGS Eastern Ecological Science Center, Leetown Research Laboratory. Optical density was measured on a multiwell plate reader (SpectraMax M4, Molecular Devices Inc., Sunnyvale, California) at 405 nm. Concentrations of the unknowns were determined from the standard curves with the Softmax Pro TM Program version 7.1.0 (Molecular Devices Inc.). Limit of detection was 1 µg/mL. Inter- and intra-assay variability was <10%.

Liver was collected in RNALater® from all fish used in this study (2013–2019). Testes were also collected in RNALater® in spring of 2015–2017 and fall of 2016 and 2018. Total RNA was extracted with an E.Z.N.A Total RNA Kit I (Omega Bio-Tek, Norcross, Georgia) from approximately 10–15 mg testes and 20–25 mg liver. A DNase treatment step was performed with RNase-free DNase set I (Omega Bio-Tek) to remove DNA contamination and all samples were eluted in 50 µL nuclease-free water. Purified RNA was quantified with an RNA BR Assay Kit (Agilent, Santa Clara, California) on a Qubit 4 Fluorometer.

Transcript abundance analyses were conducted with 50 ng of RNA/sample on the Nanostring nCounter® (Nanostring Technologies, Inc., Seattle, WA). Reproductive transcripts from a previously published liver CodeSet (Hahn et al. 2016) were analyzed (Table 2).

**Table 2:** Reproduction-related and housekeeping transcripts (asterisk) analyzed in smallmouth bass livers collected at the Antietam Creek – Potomac Mainstem site in 2013–2019

Transcript name	Transcript symbol
17-beta Hydroxysteroid Dehydrogenase	*17βhd*
40S Ribosomal Protein S12*	*40SrpS12*
Androgen Receptor α	*arα*
Androgen Receptor β	*arβ*
Choriogenin	*chg*
Elongation Factor 1α*	*ef1α*
Estrogen Receptor α	*erα*
Estrogen Receptor β1	*erβ1*
Estrogen Receptors β2	*erβ2*
Eukaryotic Translation Initiation Factor 3D*	*etif3d*
Ribosomal Protein L8*	*rpl8*
Vitellogenin	*vtg*

Testes transcripts were measured using two custom CodeSets developed from the SMB testes transcriptome (Walsh et al. 2022a). A total of 58 transcript sequences (including three housekeeping transcripts) were used for the smallmouth bass testes immune custom CodeSet (Supplementary Table 1) and an additional 43 (including three housekeeping transcripts) were included in the smallmouth bass testes reproductive custom CodeSet (Supplementary Table 2).

Transcript abundance was normalized with internal positive and negative controls and housekeeping transcripts included in each custom CodeSet using nSolver 4.0 (Nanostring Technologies, Inc.).

### Statistics

A Kruskal–Wallis one-way ANOVA was used to analyze differences in biological endpoints, including age, total length, weight, GSI, and plasma Vtg between sexes with seasons separated. A Kruskal–Wallis test was also used to analyze seasonal differences in TO prevalence and severity and plasma Vtg in males and females. For a temporal analysis of these endpoints, Dunn’s test was conducted with the package “dunn.test” (Dinno 2017) with sex and season analyzed separately. All statistical analyses were conducted with R version 4.1.1 (R Core Team 2021). The function “rcorr” in the “hmisc” package (Harrell 2021) was used to create a Spearman’s rank correlation matrix to identify associations with transcripts and biological endpoints, with sex and season analyzed separately.

To understand associations between water chemistry and biological endpoints, some of the commonly detected contaminants were used in Spearman’s rank correlation analysis. Due to different response times for the biological indicators, we used different chemical measurements depending on the response being examined. For example, to understand associations with transcript abundance, contaminant concentrations sampled within 1 week of when fish were collected were analyzed since gene regulation can occur within hours to days. For other indicators (such as plasma Vtg and GSI), the mean chemical concentration sampled within the spring season (March to within 1 week of when fish were sampled) was analyzed. This was to account for endpoints such as plasma Vtg, which changes within weeks to months. Contaminants analyzed for correlations included cholesterol, atrazine, metolachlor, simazine, fipronil, prometon, equol, formononetin, as well as E2Eq. Land-use attributes included pesticide application, % high phytoestrogen crop cover, and total nutrients from biosolids in the immediate and upstream catchment. There were not enough observations to perform correlations with the testes reproductive or immune transcripts, thus only liver transcripts were included. Males and females were analyzed separately and only in the spring (there were not enough fall observations). For all statistical analyses, results were considered significant with a *p* value < 0.05.

Lastly, a differential expression (DE) analysis of liver and testes reproductive and immune transcripts was conducted in nSolver 4.0 (using geometric means) to compare males with low, medium, and high TO severity rankings amongst each other (with testis transcripts) and to females (with liver transcripts). These analyses grouped all years with seasons separated. A cutoff fold-change value of 1.5 with a false-discovery rate (FDR) of < 0.05 was considered statistically significant.

## Results and discussion

There is increasing recognition that effects-based monitoring is necessary to understand environmental risk of chemical exposure and other environmental stressors (Dubé and Munkittrick 2001; Sanchez and Porcher 2009; Ekman et al. 2013; Schuijt et al. 2021), primarily due to the increasing (and often unmeasured) chemicals and mixture effects. Long-term monitoring at various levels of biological organization can assist in distinguishing natural variation and low-level effects of environmental stressors (Sandström et al. 2005), providing management agencies with knowledge of temporal variation, allowing them to identify trends (Lohner and Dixon 2013), recurring patterns (Flinders et al. 2015) and actions for mitigating identified risks. Integrating chemical monitoring, such as surface water and tissue analyses, with biological effects is critical for obtaining and understanding adverse effects, particularly for non-model species such as SMB. However, long-term integrated monitoring studies are infrequent due to numerous constraints, including funding, time, consistency in personnel, politics, scale, and the need for collaborative efforts (Biber 2013; Munkittrick et al. 2019). In this study, SMB were sampled over multiple years and seasons for biological endpoints important in reproduction, including gene transcript expression (molecular), histopathology (cellular), and the gonadosomatic index (tissue) in conjunction with land use and surface water chemical analyses to address concerns regarding high rates of intersex and population declines.

Data has been made available through the U.S. Geological Survey’s ScienceBase-Catalog (Walsh et al. 2022b). Twenty adult SMB were collected for each sampling effort except in 2019 when only 11 fish were sampled due to availability. In brief, mean age ranged from 2.6 to 6.4 years, lengths ranged from 252.3 to 368.6 mm, and weights ranged from 223.80 to 708.78 gm in both sexes. There was no significant difference in age, length, or weight between sexes in either season when years were combined. The mean GSI ranged from 0.42 to 0.91 in males and 0.91 to 9.01 in females and was significantly greater in females than in males in both spring and fall (*p* < 0.001). There were annual differences for both sexes in age, weight, length and GSI (Table 3).

**Table 3:** Morphometric and age data for smallmouth bass collected 2013–2019 presented as the mean ± standard error of the mean (SEM)

Collection date	Sex (*n*)	Age (years)	Length (mm)	Weight (gm)	GSI
March 29, 2013	M (11)	6.2 ± 0.3^a^	341.2 ± 7.3^a^	559.27 ± 34.35^a^	0.56 ± 0.06^a^
May 5, 2015	M (15)	4.9 ± 0.4^ab^	318.00 ± 11.79^ab^	431.67 ± 45.33^ab^	0.57 ± 0.06^a^
April 14, 2016	M (12)	3.8 ± 0.2^bc^	321.92 ± 4.94^ab^	468.25 ± 24.22^ab^	0.72 ± 0.09^ab^
October 20, 2016	M (10)	2.8 ± 0.2*	252.30 ± 11.68*	223.80 ± 31.89*	0.42 ± 0.02
May 2, 2017	M (7)	3.9 ± 0.4^bc^	285.14 ± 10.60^b^	322.86 ± 42.06^b^	0.58 ± 0.05^a^
May 5, 2018	M (12)	4.5 ± 0.4^bc^	308.09 ± 16.47^ab^	402.09 ± 74.14^b^	0.91 ± 0.10^b^
October 30, 2018	M (10)	3.8 ± 0.3*	320.00 ± 10.43*	395.10 ± 33.59*	0.48 ± 0.03
May 23, 2019	M (6)	2.8 ± 0.3^c^	282.17 ± 17.64^b^	304.67 ± 52.40^b^	0.80 ± 0.04^ab^
March 29, 2013	F (9)	6.4 ± 0.3^a^	368.56 ± 6.41^a^	708.78 ± 35.62^a^	3.85 ± 0.53^a^
May 5, 2015	F (5)	5.4 ± 0.9^ab^	304.80 ± 15.69^ab^	391.20 ± 59.58^ab^	6.94 ± 1.06^ab^
April 14, 2016	F (8)	4.8 ± 0.5^ab^	337.25 ± 4.96^ab^	532.50 ± 22.94^ab^	9.01 ± 0.57^b^
October 20, 2016	F (10)	3.5 ± 0.3	263.10 ± 12.32*	242.20 ± 29.14*	0.91 ± 0.13*
May 2, 2017	F (13)	4.4 ± 0.4^bc^	310.38 ± 21.36^b^	470.31 ± 101.78^b^	5.87 ± 1.06^a^
May 5, 2018	F (8)	3.4 ± 0.2^bc^	295.78 ± 12.60^b^	339.78 ± 44.71^b^	5.35 ± 0.60^ab^
October 30, 2018	F (10)	4.3 ± 0.5	312.80 ± 10.43*	398.60 ± 41.46*	1.50 ± 0.13*
May 23, 2019	F (5)	2.6 ± 0.2^c^	273.60 ± 17.59^b^	254.20 ± 45.01^b^	7.37 ± 1.06^ab^

Differences between years for spring samples are indicated with different lowercase letters. Since there were only 2 years of fall fish collections (2016 and 2018) a difference is indicated with an asterisk (*)

**Fig. 2:**
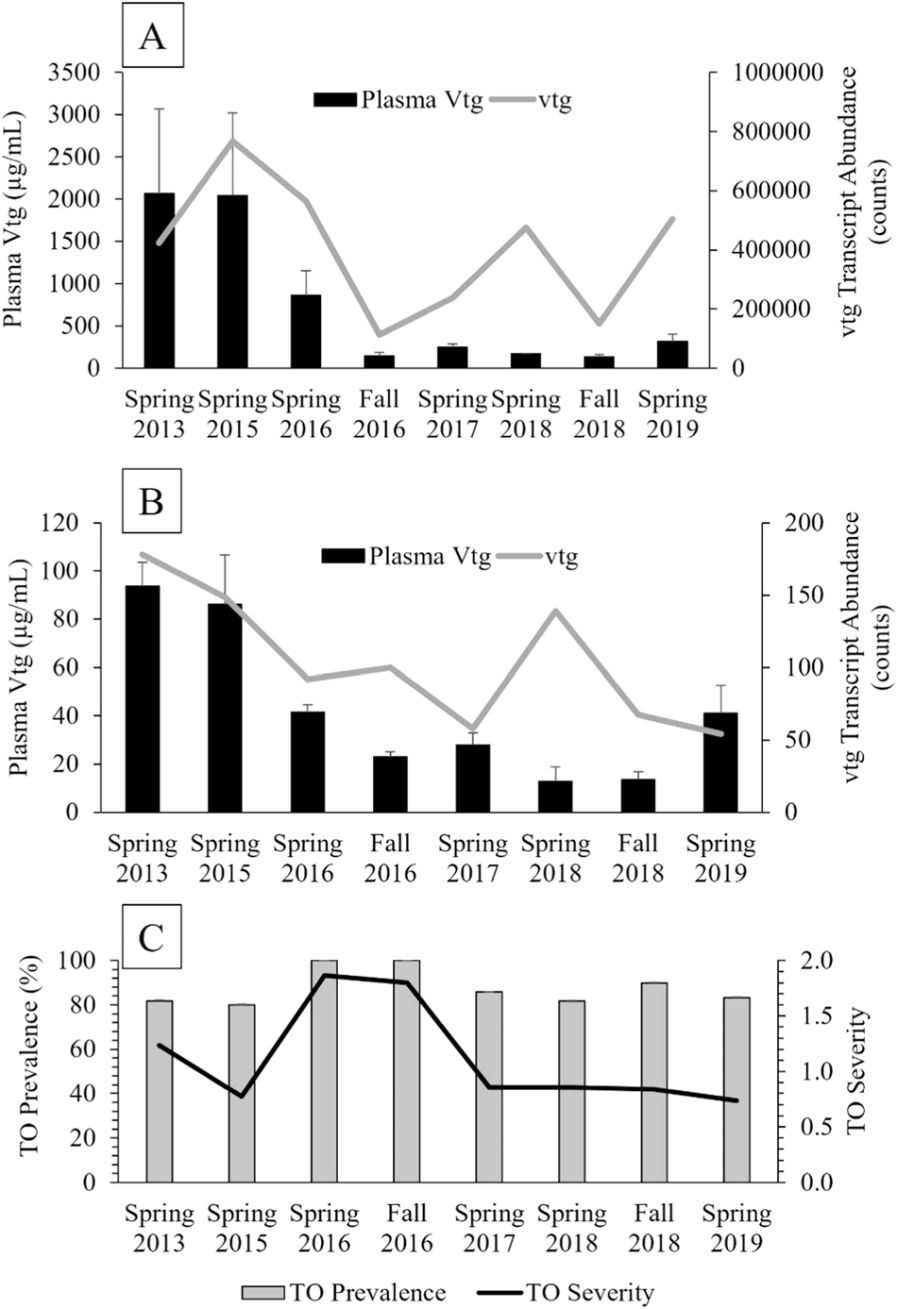
Temporal and seasonal analysis of plasma vitellogenin (Vtg) in **A** female and **B** male smallmouth bass. **C** Prevalence and severity of testicular oocytes (TO) in male smallmouth bass

Indicators of reproductive endocrine disruption with varied response times were measured, including hepatic *vtg* transcripts (response in hours to days), plasma Vtg (response in days to months) and prevalence and severity of TO (generally induced early in development). Abundance of hepatic *vtg* transcript was high in both females (Fig. 2A) and males (Fig. 2B) in 2013 and 2015. In males, abundance decreased through 2016 and 2017 but then peaked again in spring 2018.

Female *vtg* transcripts decreased in 2017 and 2018 but then peaked again in 2019. Plasma Vtg ranged from below detection (BD) – 93.72 µg/mL in males and BD – 2067.55 µg/mL in females and was significantly greater in females than males in the spring and fall (*p* < 0.001). Overall, males and females exhibited similar trends in plasma Vtg (although female concentrations were higher), with the highest concentrations in the spring of 2013 and 2015 which were reduced by almost half starting in spring 2016 (Fig. 2A, B). Prevalence of TO was high and ranged from 80 to 100% and severity ranged from 0.73 to 1.87, with the greatest severity and prevalence observed in the spring and fall of 2016. TO severity was significantly higher in the spring of 2016 than in 2015 and 2019 and significantly higher in fall 2016 than fall 2018 (Fig. 2C).

Seven other reproduction-related liver transcripts were measured to better understand the mechanisms contributing to estrogenic endocrine disruption. We evaluated the association of these transcripts with TO in males and plasma Vtg in both males and females. In females in the spring, six (*chg, vtg, erα, arα, 17βhd, erβ2*) of the eight liver-reproductive transcripts were positively associated with plasma Vtg. Four of these, *chg, vtg, erα*, and *arα*, were also positively correlated with plasma Vtg in the fall while there was an additional negative association with *arβ*. In males collected in spring, there were no liver transcripts associated with TO severity and only *chg* was positively associated with plasma Vtg. In the fall, TO severity was positively associated with *erα* and *erβ1* (Table 4), which may demonstrate the importance of estrogenic exposures during recrudescence.

**Table 4:** Results of a Spearman’s rank correlation analyses used to identify significant associations between liver transcripts and plasma vitellogenin (Vtg) and testicular oocytes in smallmouth bass

	Season	Transcript	Correlation coefficient (*r*)	*p* value
Plasma Vitellogenin				
Females	Spring	*17βhd*	0.46	0.001
		*chg*	0.32	0.028
		*erα*	0.34	0.017
		*erb1*	0.38	0.008
		*arα*	0.47	0.001
		*vtg*	0.42	0.003
	Fall	*chg*	0.81	<0.001
		*erα*	0.82	<0.001
		*arα*	0.64	0.003
		*arβ*	−0.45	0.048
		*vtg*	0.74	<0.001
Males	Spring	*chg*	0.34	0.010
Testicular Oocyte Severity				
	Fall	*erα*	0.60	0.005
		*erβ1*	0.55	0.012

In males, there were no liver transcripts associated with plasma Vtg in the fall and no transcripts associated with testicular oocyteseverity in the spring

We also examined correlations between testes transcripts and TO prevalence and severity. In the spring, only *42sp43* was positively correlated and *hsp71* negatively correlated with TO prevalence. However, multiple testes transcripts were positively associated with TO severity in the spring (Table 5).

**Table 5:** Results of a Spearman’s rank correlation analysis used to identify significant associations between testicular oocyte prevalence or severity and transcript abundance in the spring

	TO prevalence	Correlation coefficient (*r*)	*p* value
Testis Reproductive	*42sp43*	0.40	0.027
	*hsp71*	−0.40	0.027
	TO severity	Correlation coefficient (*r*)	*p* value
Testis Reproductive	*42sp43*	0.59	0.001
	*zp3*	0.50	0.004
	*up1*	0.49	0.005
	*sox11*	0.44	0.014
	*dmrt2*	0.41	0.022
	*zpax*	0.41	0.022
	*up2*	0.40	0.025
	*zp4*	0.39	0.029
	*star*	0.39	0.029
	*3bhd*	0.39	0.032
	*dmrt3*	0.39	0.032
	*cyp p450 11b*	0.38	0.034
	*ara*	0.37	0.043
Testis Immune	*afabp*	0.36	0.045
	*socs3*	0.39	0.028
	*tgfb2*	0.38	0.037
	*tollip*	−0.40	0.027

Results were statistically significant at p value < 0.05

In addition to *42sp43*, there were 15 other transcripts (12 reproductive and three immune) positively associated with TO severity, while *tollip* was negatively associated. Of these, nine had the highest abundance in spring 2016 and 2017 and the lowest abundance in the spring of 2015 and fall 2018 (Supplementary Table 3).

These transcripts were primarily female-related, including three zona pellucidas (*zp3, zp4, zpax*; Litscher and Wassarman 2018), two zona pellucida-related transcripts (*up1* and *up2*; Walsh et al. 2022b), *star* (Prucha et al. 2020), and *sox11* (Zheng et al. 2020). Additionally, two male-associated transcripts were highest in spring 2016 and 2017 and positively associated with TO severity; including c*yp p450 11b*, an androgen-related transcript previously found exclusively in male rainbow trout *Oncorhynchus mykiss* prior to sexual differentiation (Liu et al. 2000) and *dmrt3*, which was found to be male-biased in most fishes (Picard et al. 2015). Although some studies have identified a negative association of male-related transcripts with TO (Bahamonde et al. 2015; Depiereux et al. 2014) or no difference between males with and without TO (Amberg et al. 2010) most studies utilize female-related transcripts (Bizarro et al. 2014; Diaz de Cerio et al. 2012; Feswick et al. 2016), providing evidence that the use of male-related transcripts as markers of TO may not be as robust. In the fall, the testes reproductive transcripts *erα* and *inhα* were found to be negatively associated with TO severity. In rainbow trout, *erα* was significantly upregulated in the testis during spermiation and upon exposure to androgens (Delalande et al. 2015) and in Chinese tongue sole *Cynoglossus semilaevis inhα* was expressed at higher rates during spermatogenesis (Zhang et al. 2020). Thus, the downregulation of these two transcripts in the testes of SMB during recrudescence could have reproductive implications relating to spermatogenesis in the spring and may play a role in TO development. The molecular mechanisms which regulate testes development are highly conserved among fishes and changes in the regulation of these mechanisms can impact testes development over a lifespan (Delbes et al. 2022). Changes in TO severity over time may reflect biological responses to contaminant stressors associated with more recent exposures, whereas the prevalence of TO may be more indicative of early life stage effects.

Of the testis transcripts analyzed in the fall, two transcripts *ghr2* and *pgis* were positively associated with both TO severity and plasma Vtg. In the goldfish *Carassius auratus*, which is also a spring spawner, *ghr* expression in the liver was found to be associated with the seasonal regulation of plasma Vtg (Moussavi et al. 2009). And in the testis, *ghr2* has been identified in the Leydig cells and is believed to be associated with steroidogenesis during spawning (Rolland et al. 2009). Prostaglandins, including *pgis*, are known to be involved in oocyte maturation and ovulation (Tirado et al. 2017) and have been shown to be upregulated in males exposed to endocrine disruptors (Ma et al. 2012). It is possible that these transcripts are involved in different regulatory pathways associated with the induction of TO and plasma Vtg in male SMB and should be considered for use in future estrogenic endocrine disruption studies to better understand their role throughout the reproductive cycle.

While it is meaningful to discuss similar transcripts associated with both plasma Vtg and TO, it is also worthwhile to focus on transcripts uniquely associated with these endpoints as these could be useful in future research as molecular markers. Transcripts associated with only TO severity were *zp4, 3βhd*, *afabp, tgfb2*, and *tollip*. Unique reproductive transcripts negatively associated with plasma Vtg included *sox9*, *fst, fst3, grhr, inha, inhbb, erb2*, and *nobox* and eight immune transcripts, including *lyzc, c9, nadph p450*, and *preb* (which were positively associated) and *hsp71, cxcl8, tnfaip2*, and *traf2* (which were negatively associated). Generally, biomarkers are selected from exposure studies which compare control fish to fish that have been exposed to a known substance (Kar et al. 2021; Zhao and Hu 2012; Zhao et al. 2014). This is difficult to do with wild fish, especially since contaminants are often detected at “reference” sites (Abdel-moneim et al. 2015) and wild fish are exposed to chemical mixtures (Altenburge et al. 2018; Ballesteros et al. 2017; Blazer et al. 2014a; Filby et al. 2007) of which the effects are often unknown. In the case of TO, there is also some level of uncertainty with histological diagnoses since the examination of the entire testes is not feasible for large species, such as SMB. For this reason, a differential expression (DE) analysis comparing males with TO and males without TO was not conducted. However, the two transcripts associated with the prevalence of TO, *42sp43* and *hsp71* did show differences between males with low, moderate, and high severity (although they were not significant; Supplementary Fig. 1).

In particular, *42sp43* was almost three times as high in males with high TO severity than males with low severity and has been found to be a useful marker of TO in other fishes (Diaz de Cerio et al. 2012).

**Fig. 3:**
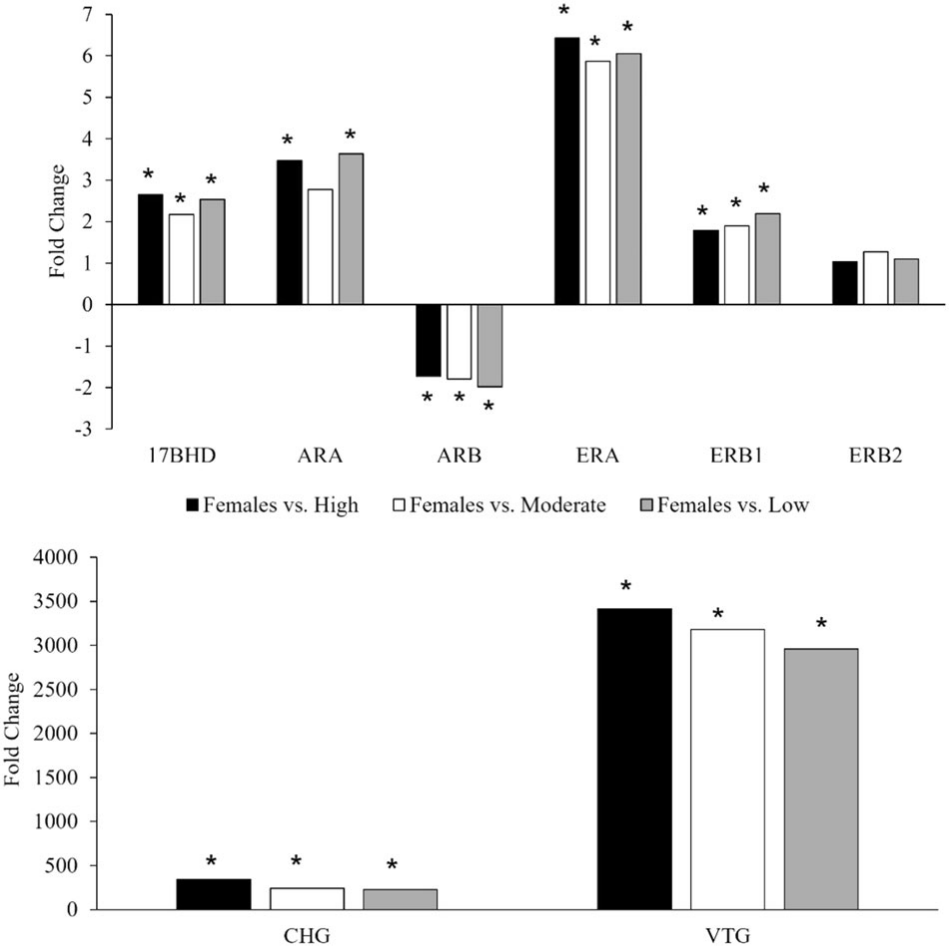
Differential expression of reproduction-associated liver transcripts in females compared to males with high, moderate, or low testicular oocyte (TO) severity in the spring. An asterisk (*) denotes a significant difference in expression from females (for example, ERB2 was not different in any males from the three rankings when compared to females). Results were statistically significant with a fold-change ≥1.5 and false-discovery rate (FDR) <0.05

The DE analysis which compared liver transcripts of females to males with low, moderate, and high TO severity revealed multiple transcripts that were significantly and differentially expressed. In the spring, although *arβ* was downregulated in females compared to all groups of males, there was a gradient effect with high TO severity males most similar to females (Fig. 3).

**Fig. 4:**
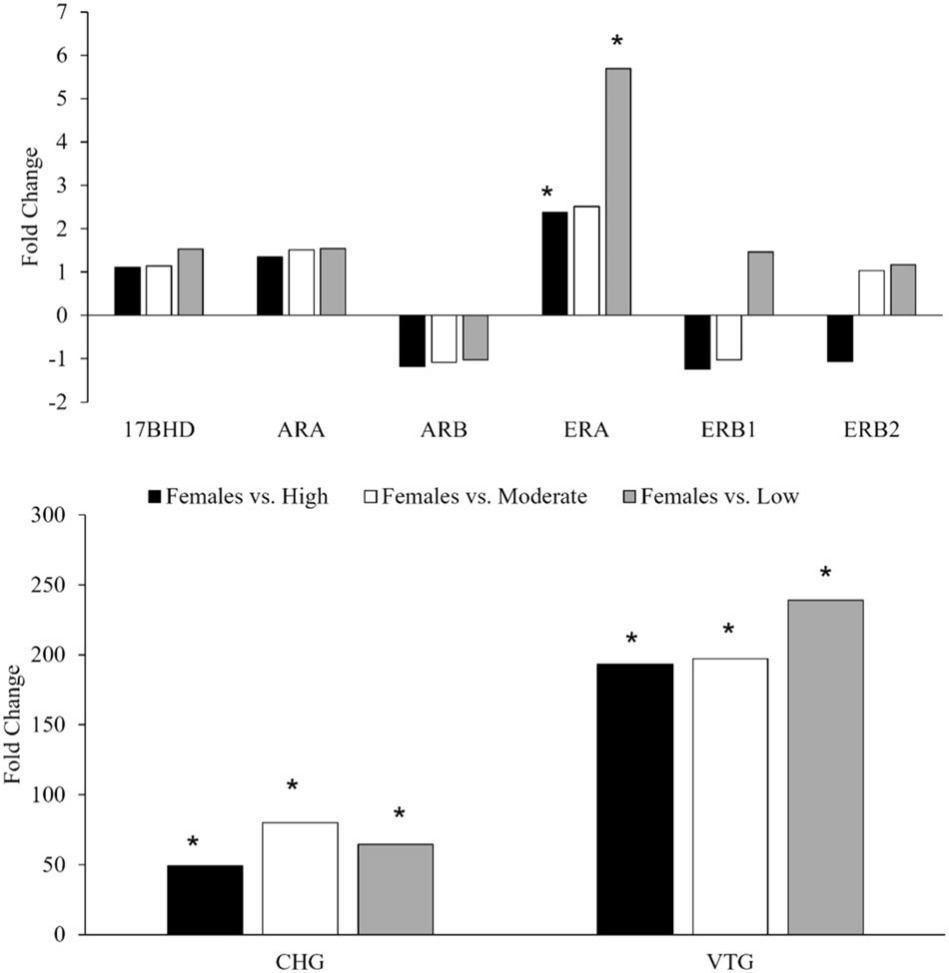
Differential expression of reproductive-associated liver transcripts in females compared to males with high, moderate, or low testicular oocyte (TO) severity in the fall. An asterisk (*) denotes a significant difference in expression from females (for example, ERA, CHG, and VTG were significantly different in males with low, moderate, and high TO severity when compared to females). Results were statistically significant with a fold-change ≥1.5 and false-discovery rate (FDR) <0.05

Regulation of *erβ1* showed a similar trend with all male groups different than females but the high TO group was most similar to females. Conversely, *erβ2* was not differentially regulated in any of the male groups. In the fall, *erα* was significantly upregulated in females compared to males with high and low TO severity but not compared to males with moderate TO severity (Fig. 4).

These results suggest that regulation of multiple transcripts, particularly in the fall, are similar between males and females. This may again indicate that recrudescence is a sensitive time for estrogenic contaminant exposures that could result in reproductive effects in the spring and more fall sampling should be considered for future studies.

The long-term monitoring of surface water chemical concentrations and land-use attributes allowed us to better identify and understand important risk factors. Analyzing contaminant exposures during early life stages such as embryos or fry is likely more meaningful than only sampling contaminants at the time adult fish are collected (Abdel-moneim et al. 2015). Male SMB sampled in 2016 (predominantly the 2013-year class) had the highest rate of TO and TO severity when compared to other years. Male bass sampled in spring 2013 also had the highest mean plasma Vtg concentrations and highest *vtg* transcript abundance. Unfortunately, surface water sampling did not begin until mid-May 2013 and phytoestrogens and total estrogenicity were not measured until 2014, limiting inferences on contaminant exposure for the 2013-year class. The surface water monitoring did document exposure to high concentrations of multiple chemical contaminants during the spring/early summer period in other years. For long-lived species such as SMB, longer time periods will be necessary to adequately document exposures from embryo to adults.

**Fig. 5:**
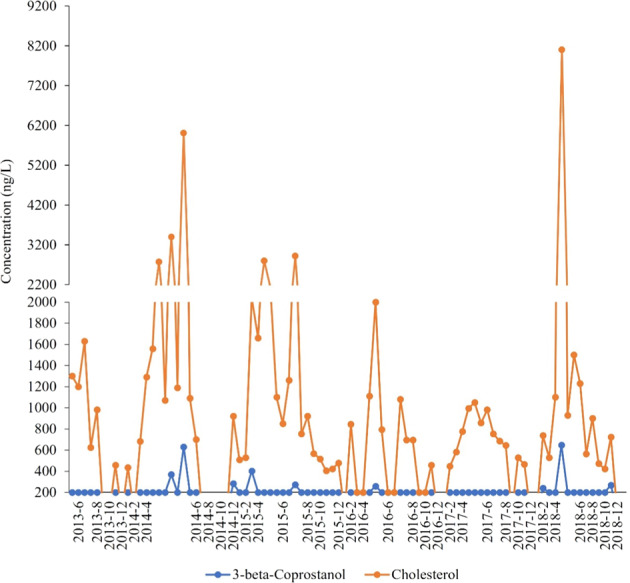
Concentrations of the two most detected sterol hormones, cholesterol and 3-beta-coprostanol sampled near the confluence of Antietam Creek and the Potomac River in Maryland, 2013–2018. Dots represent the actual sample dates

Not all chemicals were analyzed for all sampling periods and many of the chemicals had no or a low occurrence of detects and were not included in correlation analyses. Chemicals that were detected at least once are listed in Walsh et al. (2022b). Of the 20 hormones and sterols analyzed, only six hormones were detected at least once: testosterone (1 detect), cis-androsterone (3 detects), estrone (4 detects), 17-alpha estradiol (2 detects), 17-beta estradiol (1 detects) and trans-diethylstilbestrol (1 detect). Three were detected in March 2015 (prespawn) and five in early June 2016, a time when eggs and/or young fish would be exposed. Two sterols, 3-beta coprostanol and cholesterol (only measured from 2013–2018), were more commonly detected. Concentrations of 3-beta coprostanol were below 500 ng/L except for peaks in May 2014 and April 2018. Cholesterol concentrations were high throughout the study with two peaks above 6000 ng/L in May 2014 and April 2018 (Fig. 5).

Cholesterol and E2Eq were positively correlated (rho = 0.89, *p* = 0.007) and cholesterol was also correlated with *erβ2* in female bass (Table 6) suggesting it, or co-occurring compounds, may contribute to estrogenicity.

**Table 6:** Results of Spearman’s rank correlation analyses used to identify significant associations among contaminants or land use applications and biological endpoints in male and female smallmouth bass

Environmental variable	Biological indicator	Correlation coefficient	*p* value
Pesticide application: immediate	Testicular oocyte severity	0.87	0.010
	*erα* (male)	−1.00	<0.001
Pesticide application: upstream	*arβ* (male)	1.00	<0.001
	*erβ1* (male)	0.90	0.037
% High-phytoestrogen cover crop	*erβ1* (male)	−0.90	0.037
	*erβ2* (male)	0.90	0.037
	*vtg* (male)	1.00	<0.001
Formononetin	Plasma vitellogenin (female)	0.70	0.049
E2Eq	Plasma vitellogenin (male)	0.77	0.041
Cholesterol	*erβ2* (female)	0.90	0.037
Simazine	*erβ1* (female)	0.90	0.037

**Fig. 6:**
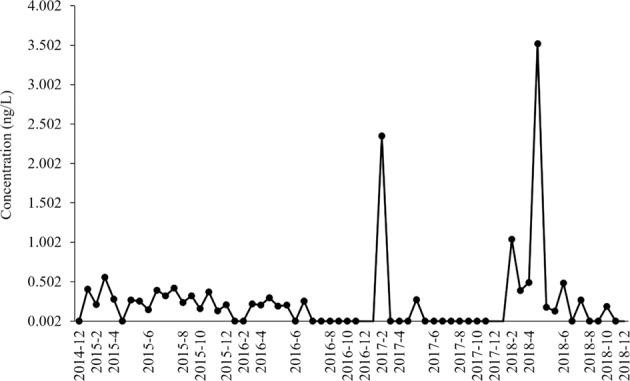
Concentrations of total estrogenicity (E2Eq; a relative equivalency measure of 17β-estradiol) sampled near the confluence of Antietam Creek and the Potomac River in Maryland, 2014–2019. In 2019 all samples were below the limit of detection and are not included. Dots represent actual sampling dates

Total estrogenicity (E2Eq) was measured from 2015 to 2019. It was generally below 1 ng/L except for peaks in February 2017 and February and April 2018 (Fig. 6).

Interestingly, E2Eq was detected above the reporting limit in 94% (16/17) of the samples in 2015 and the number of detects dropped in 2016 to only 40% (6/15) and stayed lower than in 2015 for the remainder of the study: 15% (2/13) in 2017, 69% (9/13) in 2018, and 0% (0/8) in 2019. In relation to biological endpoints, a similar trend was observed for plasma Vtg (high in 2015 and decreased starting in 2016) in both males and females. In males, E2Eq was positively correlated with plasma Vtg (Table 6).

**Fig. 7:**
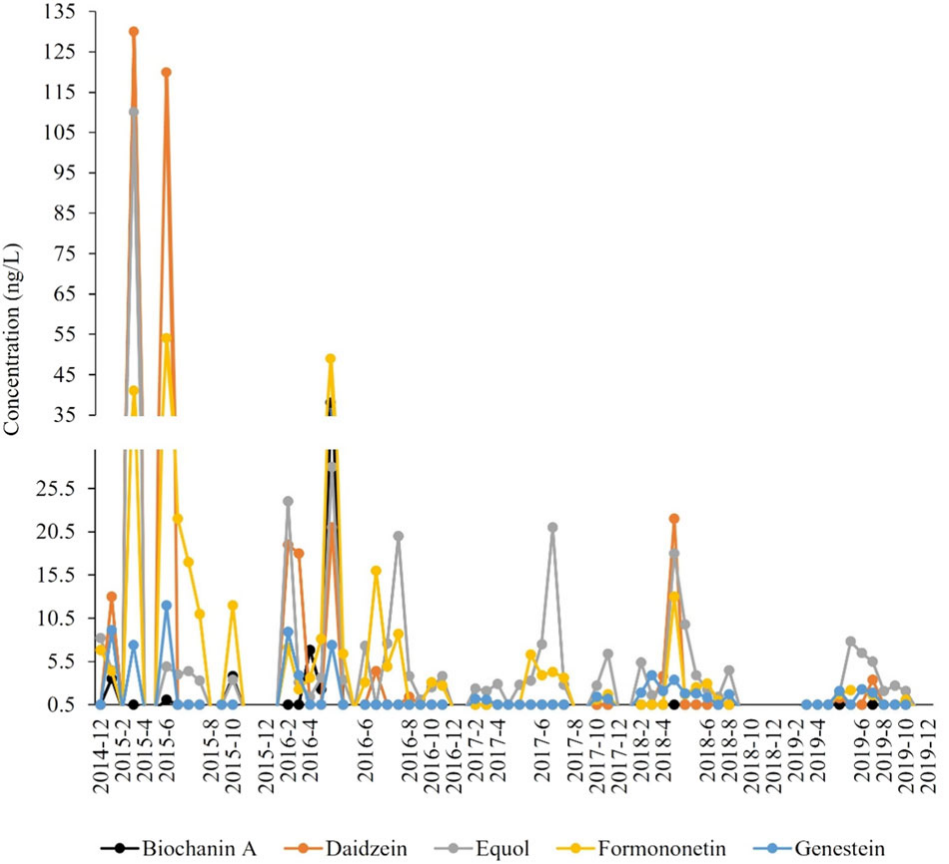
Concentrations of the five commonly detected phytoestrogens (genestein, equol, biochanin A, formononetin, and daidzein) sampled near the confluence of Antietam Creek and the Potomac River in Maryland, 2014–2019. Dots represent actual sampling dates

Phytoestrogens were measured from the end of 2014–2019. Coumesterol was not detected while the other five phytoestrogens occurred commonly throughout the study. In March and June 2015 daidzein had two peaks greater than 100 ng/L and formononetin greater than 30 ng/L (Fig. 7).

Formononetin (detected from March to within 1 week of fish sampling) was positively correlated with plasma Vtg in females (Table 6). Additionally, peaks in the springs of 2015, 2016 and 2018 corresponded with the increased abundance of four transcripts, *17βhd, arα, chg* and *erα* that also positively correlated with plasma Vtg (Supplementary Table 3). Although there is natural seasonal variation in Vtg concentrations, particularly in females, the correlations of these indicators with formononetin suggests it is a factor that needs further study.

Equol, an isoflavone metabolite, derived from daidzein and mainly occurring in the rumen of domestic animals and the human gut, enters the aquatic environment through the application of manure/biosolids (Hoerger et al. 2011). Concentrations peaked at over 100 ng/L in spring 2015, with lower peaks around 20 ng/L in the springs of 2016, 2017 and 2018 (Fig. 7). Equol induced TO in medaka (Kiparissis et al. 2003) with a dose-dependent increase from 10% prevalence at 11.5 ng/L up to 40% at 735 ng/L (Wang et al. 2020). In wild so-iuy mullet (*Mugil soiuy*) equol (concentrations ranging from 0.10 to 156 ng/L) was reported to be the most likely causal agent for intersex. Evaluation of the relative agonistic activity of equol on medaka and mullet *erα* indicated mullet were more sensitive than medaka (Wang et al. 2020). Hence, both daidzein and equol are potentially important risk factors.

Percent high phytoestrogen crop cover was highest in the immediate catchment in 2018 and in the upstream catchment in 2016. Percentages in the immediate catchment tended to increase between 2013 and 2018, while they remained more similar in the upstream catchment (Table 1). The percent of high phytoestrogen cover crops in the upstream catchment correlated with the abundance of several liver transcripts in male bass. Transcripts of *vtg* and *erβ2* were positively correlated while *erβ1* was negatively correlated (Table 6). Phytoestrogens can exhibit hormone-mimicking properties and can bind to estrogen receptors (ERs), resulting in the stimulation of ER activity (Vitale et al. 2013). In a study on rainbow trout, Leaños-Castaneda and Van Der Kraak (2007) determined that the main ER associated with vitellogenin production was *erβ*. In the liver of female SMB during the spring, *erβ1* was positively correlated with plasma Vtg (Table 4). In the liver of males, *erβ2* was associated with % high phytoestrogen crop cover (Table 6). Results from the differential expression analysis showed that *erβ2* was the only transcript not differentially regulated when compared to females. Thus, it appears that phytoestrogens may have had significant effects on plasma Vtg and *erβ* transcript regulation in the liver of both sexes in the spring.

**Fig. 8:**
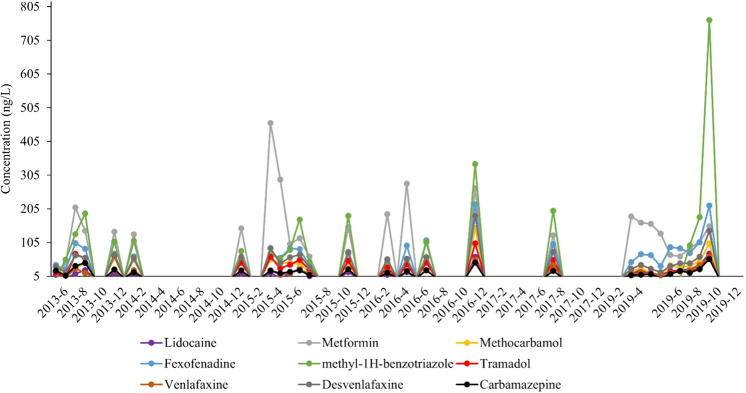
Concentrations of nine of the most detected pharmaceuticals (fexofenadine, metformin, tramadol, carbamazepine, methocarbamol, venlafaxine, lidocaine, methyl-1H-benzotriazole, and desvenlafaxine) sampled near the confluence of Antietam Creek and the Potomac River in Maryland, 2013–2019. Dots represent actual sampling dates

The pharmaceutical schedule was only measured at select time periods in 2013–2019 for a total of 27 times. Of the 107 pharmaceutical analytes, a total of 32 were detected during this study of which 66% (21/32) were detected in early March 2015. Of the analytes detected, 21 were detected 10 or more times: hexamethylenetetramine (10 detects), guanylurea (10 detects), gabapentin (10 detects), bupropion (11 detects), carbamazepine (26 detects), cotinine (14 detects), sulfamethoxazole (16 detects), trimethoprim (11 detects), lidocaine (25 detects), acyclovir (15 detects), metformin (26 detects), oxycodone (15 detects), methadone (10 detects), methocarbamol (26 detects), fexofenadine (27 detects), methyl-1H-benzotriazole (20 detects), tramadol (26 detects), metoprolol (15 detects), sitagliptin (13 detects), venlafaxine (27 detects), and desvenlafaxine (27 detects). Of the nine most detected compounds (Fig. 8), metformin (26 detects) and methyl-1H-benzotriazole (20 detects) had the highest concentrations. Methyl-1H-benzotriazole is a heterocyclic compound with numerous industrial uses which bioaccumulates in fishes and has been shown to have endocrine disrupting properties (Shi et al. 2019).

Metformin, one of the most prescribed pharmaceuticals, peaked above 200 ng/L in July 2013, March and April 2015, and April and November 2016. Metformin has been shown to induce plasma Vtg in adult fathead minnow (Niemuth et al. 2015) and intersex with early life stage exposure (Niemuth and Klaper 2015). Beginning in November 2016 the metformin metabolite guanylurea was added to the list of analytes. It was detected in all but one water sample from November 2016 through September 2019 at concentrations ranging from 193 to 1130 ng/L (Walsh et al. 2022b). The effects of guanylurea, alone or in combination with the parent compound, on the reproductive health of wild fishes is currently unknown.

**Fig. 9:**
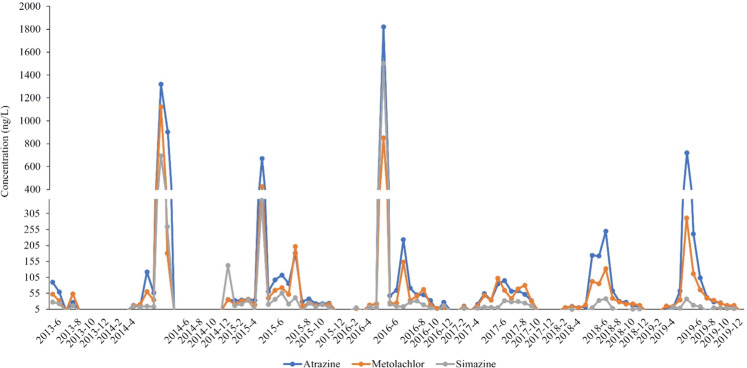
Concentrations of three of the most detected pesticides (simazine, atrazine, and metolachlor) sampled near the confluence of Antietam Creek and the Potomac River in Maryland, 2013–2019. Dots represent actual sampling dates

There were 105 current-use pesticides measured from 2013 to 2017 and only 16 were detected at least once. Of these, four (3,5-dichloroaniline, fenhexamid, carbaryl and pendimethalin) were detected once, myclobutanil twice, fipronil sulfone and boscalid three times, and tebuconazole five times. In 2018–2019 a different analytical schedule was used, and an additional 15 parent compounds were detected. Nicosulfuron, diazinon, bromacil, oryzalin, and propoxur were detected once, diuron, acetochlor, and diketonitrileisoxaflutole twice, and propiconazole and sulfentrazone three times. Atrazine, simazine and metolachlor were commonly detected throughout the study with peaks of all three above 400 ng/L in spring 2014 and above 800 ng/L, with atrazine reaching over 1800 ng/L, in the spring of 2016 (Fig. 9).

Atrazine concentrations were also above 400 ng/L in spring 2015 and 2019 (Fig. 9). Atrazine has been associated with reproductive endocrine disruption in fishes (Rohr and McCoy 2010; Kolpin et al. 2013; Leet et al. 2020) and exposure to high concentrations at key developmental periods may be of concern. However, no correlations with specific biological endpoints were observed in this study, which has been found previously (Van Der Kraak et al. 2014). Further work is required to understand effects of these complex mixtures of herbicides, particularly given the correlations with the land-use attributes below.

There was a greater percentage of agriculture and development and less forested land cover in the immediate catchment than in the upstream catchment. In the upstream catchment, wastewater treatment plants (WWTP), industrial discharges, and national pollutant discharge elimination systems (NPDES) were identified, whereas there were none in the immediate catchment (Fig. 1). The highest total pesticide application occurred in 2013 in the immediate catchment and tended to decline throughout the study period, while the highest in the upstream catchment was in 2015. Pesticide application in the immediate catchment was the only factor positively correlated with TO severity. Total pesticide application was also associated with transcript abundance of several other reproductively important gene transcripts in male SMB. In the immediate catchment pesticide application was negatively correlated with *erα*, while in the upstream catchment there was a positive correlation with *arβ* and *erβ1* (Table 6).

**Fig. 10:**
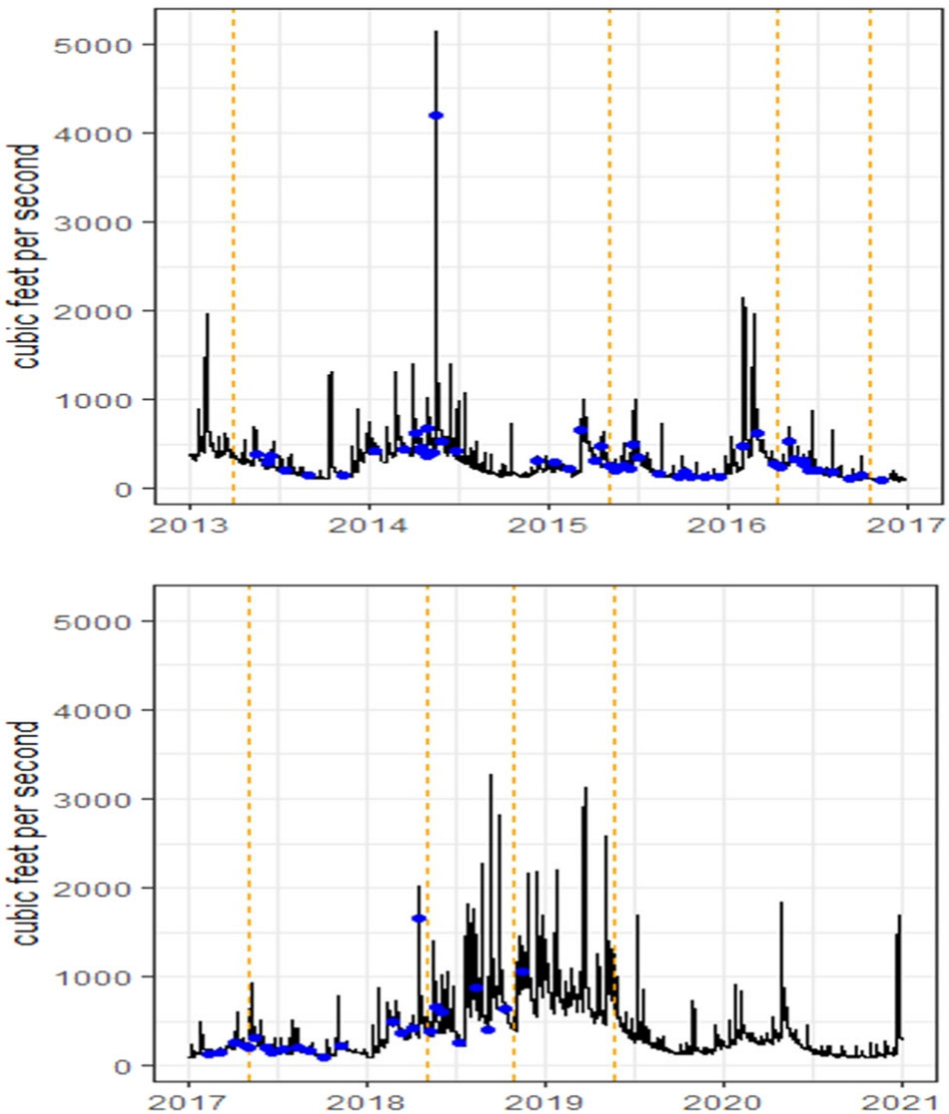
Flow (discharge) in 2013–2016 (upper panel) and 2017–2020 at the Sharpsburg gage near the Antietam Creek collection site. Blue dots indicate surface water sampling times and yellow-dash lines are sampling times for adult smallmouth bass

Discharge data was obtained from the USGS 01619500 water monitoring station on Antietam Creek near Sharpsburg, MD (39.44978, −77.73019). Flow varied considerably among the seasons and years. Peak flows were observed early spring 2013 and 2016 and late spring 2014 and 2018, while higher flows were noted from late 2018 through 2019 (Fig. 10).

**Fig. 11:**
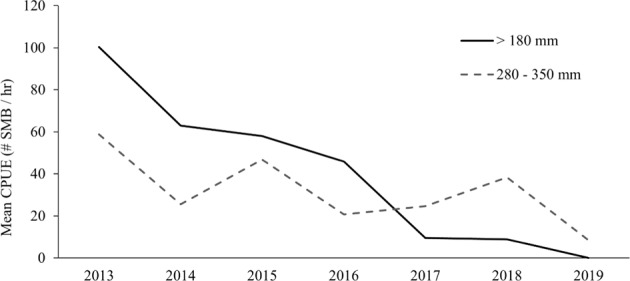
Data on the catch per unit effort (CPUE; number of fish collected per hour) of smallmouth bass collected within the size ranges of >180 mm and between 280 and 350 mm from 2013 to 2019. Data was collected and provided by the Maryland Department of Natural Resources

Peak flows often coincided with peaks of phytoestrogen and pesticide detections. The results at the cellular and molecular level suggest that endocrine disrupting effects may be contributing to population declines. Data from MD DNR population surveys conducted in 2013–2019 showed an alarming decrease in the number of SMB in the size range of 180–350 mm (Fig. 11).

In 2013 the average CPUE of fish in this size range was 159 and plummeted to less than 9 fish in 2019. Previously it was shown that SMB with TO have reduced sperm motility and abundance (Blazer et al. 2012). Studies of other fishes have shown that intersex can negatively affect fertilization rates (Jobling et al. 2002; Fuzzen et al. 2015), reproductive success of moderate to severe intersex males (Harris et al. 2011), and lead to population declines (Kidd et al. 2007). While there were years during this study with high flow events during the spring or early summer, years when flows were not excessive did not result in a greater young-of-the-year SMB recruitment, hence it is unlikely that high flows alone are responsible for the SMB population decline. At a site approximately 31 river miles upstream from this study, the same high flow events were recorded; however, the SMB population in that area has been stable (unpublished data, Michael Kashiwagi MD DNR).

In conclusion, this study provides temporal and seasonal trends in biological indicators associated with endocrine disruption. The biological indicators that were analyzed are known to be affected by contaminant exposures early in life (TO), within months (plasma Vtg), and within hours (gene transcripts). TO prevalence was high in bass sampled throughout this study, as was the rate of detectable plasma Vtg and abundance of transcripts associated with estrogenic endocrine disruption. Analysis of contaminant exposures during early development showed that TO development may have been affected by pesticide application within the immediate catchment. Additionally, the percentage of high phytoestrogen cover crops and pesticide application correlated with a number of gene transcripts important in reproduction. Peak concentrations of multiple chemicals contributed to the complex mixtures potentially affecting bass during the spring (spawning), early summer (early juvenile development) and fall (recrudescence). However, to understand exposure during critical life stages in a wild population consisting of multiple year classes, longer term studies at multiple sites will be needed. The data obtained provides environmentally relevant concentrations and identification of complex mixtures for in vivo and in vitro exposure studies on SMB. It also identified potential molecular markers to better understand risk factors and adverse outcome pathways.

While many contaminants were measured in the surface water samples, it is important to recognize that numerous chemicals not measured are part of the SMB exposome. These include polyfluoroalkyl substances (PFAS) detected in plasma (Blazer et al. 2021a) and mercury (Willacker et al. 2020) in muscle/liver from this site. Temperature, another climatic factor, should also be considered to better understand its relationship with changes in reproductive endpoints. In previous studies (Blazer et al. 2007, 2010, 2012) a lack of histopathological findings in ovaries and the observance of intersex in males led us to further investigate gene expression in only the testes and not the ovaries; however, some of the contaminants in this study can have a negative impact on female reproduction and future studies should include ovarian transcript abundance analysis. Lastly, the study documents the temporal changes in exposure to complex mixtures of chemicals that may act synergistically, additively or antagonistically and these interactions need to be addressed in laboratory studies.

## Supplementary information

Supplementary Figure 1

Supplementary Table 1

Supplementary Table 2

Supplementary Table 3

## Data Availability

Data are available through the U.S. Geological Survey’s ScienceBase-Catalog at https://www.sciencebase.gov/catalog/item/627a76f3d34e8d45aa6e4e1a.
