# Supplementary material for: A case study: temporal trends of environmental stressors and reproductive health of smallmouth bass (Micropterus dolomieu) from a site in the Potomac River Watershed, Maryland, USA
Source: Ecotoxicology. 2022 Dec 1;31(10):1536–53. doi: 10.1007/s10646-022-02605-8 (PMC9729326; doi:10.1007/s10646-022-02605-8)
Supplement: Supplementary file 2 — Supplementary Table 1 [file 10646_2022_2605_MOESM2_ESM.docx]

A Case Study: Temporal Trends of Environmental Stressors and Reproductive Health of Smallmouth Bass (*Micropterus dolomieu*) from a Site in the Potomac River Watershed, MD, USA

Heather L. Walsh*^1^, Stephanie E. Gordon^1^, Adam J. Sperry^1^, Michael Kashiwagi^2^, John Mullican^3^, and Vicki S. Blazer^1^

^1^U.S. Geological Survey, Eastern Ecological Science Center, Leetown Research Laboratory, 11649 Leetown Rd., Kearneysville, West Virginia 25430, USA

Corresponding author: *hwalsh@usgs.gov

^2^Maryland Department of Natural Resources, Fishing and Boating Services, 10932 Putman Rd., Thurmont, Maryland 21788, USA

^3^ Maryland Department of Natural Resources, Fishing and Boating Services, 20901 Fish Hatchery Rd., Hagerstown, Maryland 21740, USA

ORCID ID:

Heather L. Walsh 0000-0001-6392-4604

Stephanie E. Gordon 0000-0002-6292-2612

Adam J. Sperry 0000-0002-4815-3730

Vicki S. Blazer 0000-0001-6647-9614

**KEYWORDS**

Reproductive endocrine disruption, testicular oocytes, plasma vitellogenin, contaminants, land use, long-term monitoring

**ACKNOWLEDGEMENTS**

We would like to thank the electroshocking crew from Area 7 of the Western Region of the Maryland Department of Natural Resources for assistance in the collection of smallmouth bass used in this study. We also thank the graduate students and technicians who have helped provide field, histology, and laboratory assistance. Any use of trade, product, or firm names is for descriptive purposes only and does not imply endorsement by the U.S. Government.

| **Transcript Name** | **Transcript Symbol** | **100 bp Probe Sequence** |
| --- | --- | --- |
| 5-hydroxytryptamine receptor 2B | *5-ht2br* | TTTCTACCGCATCCATCATGCACCTATGCGCCATTTCACTGGATCGCTACATTGCCATTAAGAAGCCAATCCAACACAGCCAGTACAAATCCAGAGCCAA |
| 40S ribosomal protein S18* | *rps18* | GAGAGCTGACTGATGAGGAGGTTGAGCGTGTGGTGACCATCATGCAGAATCCTCGCCAGTACAAAATCCCAGACTGGTTCCTCAACAGGCAGAAGGACGT |
| B-actin* | *actb* | GTACGTTGCCATCCAGGCTGTGCTGTCCCTGTATGCCTCTGGTCGTACCACTGGTATCGTCATGGACTCCGGTGATGGTGTGACCCACACAGTGCCCATC |
| Complement component C6 | *c6* | GCCACAGAGACCTGCCAACAGTTTCCTCAGGAAAGCAAAGCAATATTATAAATTTGGTGAGGATGAGGAGTTCGTGTGCTTCACTGGATTTGACTTGGAG |
| Complement component C7-like | *c7* | GTACTTACAGATGATGGAGCCTCAGTGCTTCAGTCTTTCTGTGACTCCACCAAAGACATGTGGGCCGCCTCCAAACCTCAGGAATGGATTTATTCAGAAT |
| Complement component C8 alpha chain | *c8a* | CAGGCCAGCTAACCCTTAAAGGAGCTCGTCCTGAGCTGAACACTGAGATAGTGATCGGGGAGACGGACTATGACTCCTATGCAATCATGTACTATCAGAA |
| Complement component C9 | *c9* | AAAAACGGGAAATCTGGTGGCGAATATGAGTTGGTCTATGTTCTCAACCAGGACACCATCAAGGAAAAAAATCTGACAGAGAGAAGTGTTCAAAACTGCG |
| C-X-C motif chemokine 11-like (I-TAC) | *cxcl11* | CTCTCATCACTGATGTCAAGGAGATTGATCCTCTTCCATATTGCAACAAGAAAGAAGTCATTGTCACACTGAGAGATAACAGCCAAAGGTGTCTTGACCC |
| Cyclooxygenase 2 | *ptgs2* | AAGACAATTCATTCCGGACCCGCAGGGCACCAGTCTGATGTTTGCATTCTTCGCGCAGCATTTCACCCACCAGTTCTTTAAATCCGATATGAAGAAAGGG |
| Cytochrome P450, family 3, subfamily A | *cyp3A* | TTCGGCACTATGCTGGCATATAAAAAGGGATTCATGCACTTTGATTCGGAGTGCTTCAAGAAATATGGGAAAACATGGGGCATTTTTGATGGCCGTCAGC |
| Deoxynucleoside triphosphate triphosphohydrolase SAMHD1-like | *samhd-1* | CAGCCAGAACTCCTCATCTCTCGCAGAGACATCCTTTGTGTGCAGATCGCCGGGCTCTGCCATGACCTTGGACATGGGCCATTTTCCCATATGTTTGATG |
| Eukaryotic translation initiation factor 3D* | *eif3d* | ACATGACTCAGTTCAACATGCAGACGCTACCTAAGAGCGCCAAGCAAAAGGAGAGGGATCGTATGCGCCTGCAGAAGAAGTTCCAAAAGCAGTTTGGTGT |
| Fatty acid-binding protein adipocyte-like | *afabp* | ACGTGATAAAACCGAAGCTGGTCATCAGTGTGGATGATGCTGGGGTTATTTCAATGAAGTCTGAAAGCACGTTCAAGACCACTGAAGTCAAGTTCAAGCT |
| G protein-coupled receptor 176 | *gpr176* | CCATGCCCGCTCCCTGGGCCACATGGTGTACATTTTGATCTACAACGTCACCACGGTGATCCTTCCTCTCGCTCTGGTCTTCCTCTTCATGCTGCTCATC |
| Growth hormone receptor-2 | *ghr2* | GTCGACCTTGTGAAAAGCACACATCGCTCGCTTTATGGGCTTCAAACTAACATCAATCACGAGATTCAGGTCCGGTGCAAAATGCTTGGTGGGAAAGAGT |
| Growth hormone receptor-like | *ghr-like* | AGAACGACGCCTTAATGGTCCCTTGTGACCTGACACCTTTGGCCGCTTCTCATCAGTTCACCCAAAACCCTTCGTTGTACATGCAGAGCGTGTCGCCCTA |
| Growth hormone regulated TBC protein 1 | *grtp1* | CCCAAACTGGTAGAGACCATCTGCACAGACTTGAACAGAACATTTCCAGACAACATCCAGTTCCGCAAGACATCCAACCCGTGTCTGCAAAAACCTCTGT |
| Heat Shock Protein 71 | *hsp71* | TCATTGCATTGTTTGCGACACAACTGAGGAGCAACTTGTTGGCACAGTTGAGCTGTCAGTTCACATGTAAAGTGTCTGGTATACTGAGAGGTCACTGCCT |
| Hepcidin 1 | *hep1* | CAGTGACACTCGTGCTCGCCTTTATTTGCATTCTGGAGAGCTCTGCCGTCCCATTCACCGGGGTGCAAGAGCTGGAGGAGGCAGGGAGCAATGACACTCC |
| Interferon regulatory factor 3 isoform X1 | *irf3* | CTCCATTCCCTGGAAACATGGTTTAAGACAGGACTCCTCAAACTCTGACATTCTCATCTTTAAGGCGTGGGCCGAGACGAGTGGCAATGGCCGGGCTCAG |
| Interferon-stimulated gene 15 | *isg15* | TCTAAGGGTTTTTCTAGAGGACGCCATTACTGGGAAGTCCGACTGAGCAGCAGCAACTTCATTGGCATTGGCTTGGCTTACAACAGCATTGACCGCAAAG |
| Interleukin 15 | *il-15* | TGAGTACATCTATGTATGCTGCTTCTGTGCCTGGTACAGACATTGTACAGACTTGCTTGGAGAAGCTAAAACACACCATTGAGAAATCTGACGCTATGCT |
| Interleukin 8 | *cxcl8* | ACATGAAGAGCAGCAAAGTCATTGTCACCTCTATCGTGGTGCTCCTGGCCTTCCTGGCCATCAGTGAAGGGATGAGTTTGAGGAGCCTGGGTGTAGAGCT |
| L-amino-acid oxidase isoform X1 | *il4i1* | CACTGATAGAGATGCTGTACATACAGTCAGACATCAATGACAACACTGAGTACTTTGAAGTGACTGATGGCTTTGACCACCTCCCAATGGCTTTCTACCA |
| Latent transforming growth factor beta binding protein 2 | *ltbp-2* | ATATGAGTACGGTTATGAGGGGCCAGACGATCCTGTGGAGCCTTTTGTTTCTCCATACTTTGACAGTTATGGAAACCCTGTGCGACCCTATGACATTCCT |
| Latent transforming growth factor beta binding protein 3 | *ltbp-3* | GTGAACCTGCCCGGTTCTTACAAATGTGAATGTCACATCGGCTTTAGGAGCAAGTCACACCGTCACCCAGCCTGTGAAGACATAAACGAGTGTTTGAATC |
| Lysozyme C-like | *lyzc* | TGGTGTAACAATGGCCGCACCCAGTCTGCGAATGGATGCAACATCAATTGCAGCGAGCTTCTGAACGGCGATATCAGCGTGGCAATCAACTGTGCCAAAC |
| NADPH oxidase1 | *nox1* | CCCTGAGGAGGACTTTTTCAGCGTCCACATCCGCTCGGCCGGGGACTGGACGGACAAGCTCATCAGCATCGTGCAGAAGCTACCAGAGGGATCGCAGGGA |
| NADPH-cytochrome P450 reductase | *nadph p450* | GAAAATATGTTGATAAAAGGCTGGAAGAACTTGGGGCCAAGCGCATCTTTGACCTTGGTTTAGGAGATGATGATGGCAATCTGGAAGAGGACTTTGTTTC |
| Prolactin receptor | *prlr* | CTGGTTGGATCACTCTCATCTACGAGCTCCGCGTCAAGTTAGAGGAGGAAAATGACTGGGAGATGCACCTTGCAGGCCAGCAGAAGATATTTAACATTTT |
| Prolactin regulatory element binding | *preb* | AAAGCACATGATGGTGAGATTGAAGACTTGGACACGAGTCCAGGGAACAAGCACCTGGTGACTGTTGGCCGGGATTTTGCCTGCAGTGTATGGAGTGGCA |
| Prostacyclin synthase | *pgis* | GAACAGAACAACAACGGCACAGATCCCTCAAGTGTCTACAAGGAGTACAAGAAGTTTGACGACCTCTTAACCAAAATGGCAAGAGGCACACTGAAGTCAG |
| Prostaglandin E synthase | *ptges* | TGCCTGTGGCCCGGCTTCACTTCCTGGTCTTCTTCATGGCTCGCATTCTGCACAGCATTGCCTACCTGTTTGCCCTGCAAGCACCAACACGCTCACTGGC |
| Selectin P ligand | *selplg* | GCCACGTCTTTGCCGCAGTATACCTCCACCACTAAACCTGAAGTTACATCCACGTTATCTTCCACAACCTCTCCTGCTGATTCAACTCAGGCTCCAGTCT |
| Suppressor of cytokine signaling 1 | *socs1* | GTATCTGACCCACTTCCCCACCTTCTCCTGCAAGGAGGACTGTGTGATCATCACAGACACAGCGTCCAAGCTCGAGCGCAGCTCCTTCTACTGGGGCCCT |
| Suppressor of cytokine signaling 2 | *socs2* | CCACCAACCTGCGGATCGAGTACAAACACGGCAAATTCAAGCTGGACTCGGTGGTTCTGGTGAAGCCCAAGCTGAAGCAGTTCGACAGCGTGGTCCACCT |
| Suppressor of cytokine signaling 3 | *socs3* | CCCACTTTGACTGTGTCCTCAAGCTGGTCCATTACTACATGTCTCAGAACAAAGGGAACACTCGCAGTGGGAATATCTACTACATTTACTCTGGCGGGGA |
| Suppressor of cytokine signaling 5 | *socs5* | GTGGGAACCGTAGGGACAGACGTTATGGAGTGTGCTCCATCCAGGACATGAGCGACTCTGTATCTGGAGGACGCAGTCTGAATGCTCGGTCTTTGCGCCA |
| Suppressor of cytokine signaling 6 | *socs6* | CGGAAAGAATTTCACGATTTCCAGATGGAAGGGCTCTTTCAGGACCAAGCAGAATCCTTAAAGAATCTCCAGCAGCCTCAAAACGGTGAGCTGCATCTAA |
| Suppressor of cytokine signaling 7 | *socs7* | CACTACAGAGGTACGTTCAGCCTCTGGTGTCACCCTAAGTTTGAGGATCGCTGTCACTCTGTGGTGGAGTTCATTGAACGAGCTATCATGCACTCCAAGA |
| T-cell receptor | *tcr* | GCGAAGAAACTTTGGAGCTAGATCCGACAGTGAACGGCATGACTCTGACCATCACGATCCTCAGGCTGATCTTTTTCAAGACCGTCGTCTTCAACATCGT |
| TNF alpha induced 3 | *tnfaip3* | CCGCCCTTCTCCGTCTCCCAGCTCTCCCTCATTGAGATCCGTTGTGCCACACCACGATGCACCTTCTATGTCTCTGTTGACACACAGCCTCATTGCCATG |
| TNF receptor-associated factor 2-like | *traf2* | TCTGCAGCAAATTCAGAACTCCGTGTCGATTTCATGTCGTGGGATGTGATATGTCTGTGGAGAAAGAAAAGATTCATGACCATGAGCGTGCCTTTGCCTA |
| TNF receptor-associated factor 3 | *traf3* | GCCAAACCGAGTGTGGACACCGCTTCTGTCAGAGCTGCATCCACGACATCCTCAGTCATCCAAACCCGGTATGTCCAGCTGACATGGAGCCTCTGTTCAA |
| TNF receptor-associated factor 5-like | *traf5* | CGGAGGTGGACTGTCCCAACAACTGCTCCCAAAAGGTTCCCAGACACAAGCTGACGGAGCACAGAGACTCGTGTCCTGAGGTTCACGTCGCCTGTTCTTA |
| TNF receptor-associated factor 6 | *traf6* | GCCTGTACTGTGAGATGGATCTCATCAGAGACCAGATGGAATCTCATTGTGATACAGATTGCCCAAAAGCACCCATCGCCTGTAACTTTAGCACCTTTGG |
| Toll interacting protein | *tollip* | ATGATCAACCTGGTGATGTCTTTTGCTTCTTTACCAGCAGGGATGATGACACAGCCTGTTGTTCTAATGCCCTCTGTGTACCAGCAAGGAGTCGGATATG |
| Toll-Like Receptor 13 | *tlr13* | TAAAACTGAGTGACAATGACATTACAGTGATCAACGAGACGGTCTTCCAGTCTCTCCCTGCACTCACATATCTGGACCTGGACAATAATCCTTTCACCTG |
| Transforming growth factor beta 1 | *tgfb1* | AATAACACAGAGAACACCAAGACCTCCAAAAGCATCCCGATGTTCTTCAGCATCTCTAACATACGGGAAAGTGTGGGGGATTCCAGTTTGCTGACCAGTG |
| Transforming growth factor beta 1 induced trasncript 1 | *tgfβ1i1* | GCATGCTGGGGCTGCTTCAGTCTGACCTGAGCCGACAAGGAGTTCAAACATCCTCCAAGGGAAACTGTTCAGCTTGTCAAAAGCCAGTAGTAGGACAGGT |
| Transforming growth factor beta 3 | *tgfβ3* | CACTTTCAGGCCTAATGGTGACATTATTGAGAATGCCAATGAGGTGCTGGAAGTCAAGTTCAAAGGTATGGATCCGGACTATGACGTGAAAAAACCCAAG |
| Transforming growth factor beta receptor 3 | *tgfβr3* | CAACACTCTGCCGTTCAACAGCTCTTCGCGCCAGGCCTTCTACACCGTTTCACAAAATCAGCAAGTCTTCGTGGAGGTCACATCAACCGCATCTGATCCG |
| Transforming growth factor beta receptor associated protein 1 | *tgfβrap1* | GCTTGTCCTCACAGAGGGAGCACAGCAAAATATTCCCAAAGACAAGTTCCAGATCTTGCACAAAAGAATCCTCCAGCAGGCAGGTTTTATACAGTTTGGC |
| Transforming growth factor beta receptor-associated protein | *tgfβrap* | TACAAGGAGCTGCAGAAGGTCATCACTTGCCTGGTTGGATTTGTTCATTTTTATCAGCAGGGCTTTTCCGAGGCCAGAAATCTTTTCATCACAGGTGAGC |
| Transforming growth factor beta-2 proprotein-like | *tgfβ2* | CTCCACCCAACGTTATATTGACTCCCGCACCGTTCAGCCAAAGGCGAAGGGAGCCTGGATCTCGGTTGAGGTCACCGAAACCATCAAGGACTGGGTGTCA |
| Tumor necrosis factor alpha induced 2-like | *tnfaip2* | CACAAGGCCCAGAACATAGCTTCCCAACTGACAGATTTAATGCAGAGGTTCAGTATTTTCCTAAACGAGGTCATCAAGCAAAACAAACCGAACAGCAAGC |
| VPS39 subunit of HOPS complex | *vps39* | GGCAAATATTGTGTACGTTGCCAGCAACCACTTTGTGTGGCGCCTTGTGCCCGTGTCAATAGCCAGCCAGATCCGACAGCTTCTTCAGGACAAGCAGTTT |
| Zinc-binding protein A33-like | *zbpa33* | CGATGTTCGCAGAGACAAAGACAAAATGGATACCATAAAGAAAGTCGTGGCAGATATGCAGGCTCGCGTCAAAGGCGAATTCAGAGTTCTACATCAGATC |
