# Supplementary material for: A case study: temporal trends of environmental stressors and reproductive health of smallmouth bass (Micropterus dolomieu) from a site in the Potomac River Watershed, Maryland, USA
Source: Ecotoxicology. 2022 Dec 1;31(10):1536–53. doi: 10.1007/s10646-022-02605-8 (PMC9729326; doi:10.1007/s10646-022-02605-8)
Supplement: Supplementary file 3 — Supplementary Table 2 [file 10646_2022_2605_MOESM3_ESM.docx]

A Case Study: Temporal Trends of Environmental Stressors and Reproductive Health of Smallmouth Bass (*Micropterus dolomieu*) from a Site in the Potomac River Watershed, MD, USA

Heather L. Walsh*^1^, Stephanie E. Gordon^1^, Adam J. Sperry^1^, Michael Kashiwagi^2^, John Mullican^3^, and Vicki S. Blazer^1^

^1^U.S. Geological Survey, Eastern Ecological Science Center, Leetown Research Laboratory, 11649 Leetown Rd., Kearneysville, West Virginia 25430, USA

Corresponding author: *hwalsh@usgs.gov

^2^Maryland Department of Natural Resources, Fishing and Boating Services, 10932 Putman Rd., Thurmont, Maryland 21788, USA

^3^ Maryland Department of Natural Resources, Fishing and Boating Services, 20901 Fish Hatchery Rd., Hagerstown, Maryland 21740, USA

ORCID ID:

Heather L. Walsh 0000-0001-6392-4604

Stephanie E. Gordon 0000-0002-6292-2612

Adam J. Sperry 0000-0002-4815-3730

Vicki S. Blazer 0000-0001-6647-9614

**KEYWORDS**

Reproductive endocrine disruption, testicular oocytes, plasma vitellogenin, contaminants, land use, long-term monitoring

**ACKNOWLEDGEMENTS**

We would like to thank the electroshocking crew from Area 7 of the Western Region of the Maryland Department of Natural Resources for assistance in the collection of smallmouth bass used in this study. We also thank the graduate students and technicians who have helped provide field, histology, and laboratory assistance. Any use of trade, product, or firm names is for descriptive purposes only and does not imply endorsement by the U.S. Government.

| **Transcript Name** | **Transcript Symbol** | **100 bp Probe Sequence** |
| --- | --- | --- |
| 17-beta hydroxysteroid dehydrogenase | *17βhd* | CATCCTCAACATCTCGTCTGCCAGTGGGATGTACCCTGTTCCTCTCCTCACTGTCTACTCTGCCTCCAAGGCGTTTGTGGACTTCTTCTCCCGAGGACTG |
| 3-beta hydroxysteroid dehydrogenase | *3βhd* | AACTTTTAACTAGAGTCTGGAGGACTGTAGAGGCGACACAAAGTTGAATGTTTTCGAGGGCGACATCAGAGACGGTGATTTCCTGAGAAAACTTGTCGTG |
| 40S ribosomal protein S18* | *40srps18** | GAGAGCTGACTGATGAGGAGGTTGAGCGTGTGGTGACCATCATGCAGAATCCTCGCCAGTACAAAATCCCAGACTGGTTCCTCAACAGGCAGAAGGACGT |
| Aromatase | *cyp19a1a* | GCTGGAGATGGTGATCGCAGCACCAGACACTCTGTCCATCAGCCTCTTCTTCATGCTGCTGCTCCTCAAACAAAATCCAGATGTGGAGCTGAAGCTGCTA |
| Beta-actin* | *βactin** | GTACGTTGCCATCCAGGCTGTGCTGTCCCTGTATGCCTCTGGTCGTACCACTGGTATCGTCATGGACTCCGGTGATGGTGTGACCCACACAGTGCCCATC |
| Cytochrome P450 11B | *cypp45011b* | GAAGCCAGCTGCCATGTGCTCTACGGGGAGCGTATTGGCCTCTTCTCCTCTTCTCCCTCCTTGGAGTCGCAGAAGTTCATCTGGGCCGTGGAGCGGATGT |
| Dehydrogenase reductase SDR family member 11 | *drsdrfm11* | TTAGCCTTGTCTATCTGCACCCGTGAGGCATACAAATCAATGAAGGAAAGGAATGTGGATGATGGCCACATAATCAATATTAACAGTATGGGTGGGCACC |
| Doublesex and mab-3 related transcription factor 1 | *dmrt1* | GCTGGAGCAGATTGCTTGTTCTCTGTGGAGGCACGATCTCCGACACCTACCAGTACCTCCGCTCCTTCTCTTGTTGTTGCAGGGAGTCGCTCGGCGGCGT |
| Doublesex and mab-3 related transcription factor 2 | *dmrt2* | AGGACGATTTATCAGCGGCATATTCGACCGTCCACCATGCTCGCCAAAAGCATCCTCGAAGGATACTGTCCGGTGCAGTCTGATCCATTCCTGGCGTCCA |
| Doublesex and mab-3 related transcription factor 3 | *dmrt3* | CAGCAGGCCAACGAGAGTCTGGAGAGCCTCATCCCGGAGTCACTCAGAGTGCTGCCCGGTATCGGTATATCCGGAGCCAGCGAGGGGAACCAGGGAGCCC |
| Eukaryotic translation initiation factor 3D* | *etif3d** | ACATGACTCAGTTCAACATGCAGACGCTACCTAAGAGCGCCAAGCAAAAGGAGAGGGATCGTATGCGCCTGCAGAAGAAGTTCCAAAAGCAGTTTGGTGT |
| Follistatin | *fst* | GGTGGGAGTGGATGAGGATATCTCCTCTCTGTTTGTTGAAGATTCGGCCAAGAAGACCCTTGCAAACATTCTTTGGAGAGAAGAAGGTTTGAGTGTGGGG |
| Follistatin 3 | *fst3* | GCATGGGACACCCGGACCTGGAGGTCATGTACCAAGGAGACTGTAAAAAGTCGTGCTCCAATGTGGTGTGCCCAGGTACCCACACCTGTGTGACGGACCA |
| Gonadotropin releasing hormone receptor | *grhr* | CTGTAGCGTGGTGCATGAGTGTCGTGCTGTCAGTCCCTCAGATATTCCTTTTTCACAACGTAACCATCATTCATCCCAAGGACTTCACTCAGTGTACCAC |
| Homeobox protein NOBOX | *nobox* | GTTAGAGCATTTAGAGGCCTTGTTCCAGGAGGACCACTATCCTGACGCAGAGAAGAGGAAAGTCATCGCTGCTTCAGTTGGTGTCACACCTCAAAGAATT |
| Inhibin alpha | *inhα* | ATCTGCCCACTTCTGGTTCTATGCAGGCAAAGGAGCCACATCCAACTCCTCTGCCCAGCTGTTCATTCTCACTTCAGAGCAGAAGCTACTTCAGGCGGCA |
| Inhibin beta b | *inhβb* | TCATCAGCTTTGCGGAGAAAGATGATATGGTGACATCCAAGTCCAGCCTCTTCTTCCAGATCTCCAGTGAGGGGAACCAGAACCTGCATGTGACGCAGGC |
| Insulin like growth factor binding protein 1 | *igfbp1* | CCCTGTCATGTTGAGCTACAGAGAGCCTTGGATAAGATTGCCAAATCCCAGCAGAAATTGGGAGACAAATTAACCAGATTCTACCTCCCTAACTGTGACA |
| Insulin like growth factor binding protein 2A | *igfbp2a* | ATGACCAAAAACCTCATGCCTATAACAATGCTGTCGTACGCGGGCGTCAGCTTGCTAATCCTCTCCGCGTCTCTCGCCGGTGCCTCCCTGGCCGAGATGG |
| Insulin like growth factor binding protein 3 | *igfbp3* | ACCGGGGCAAACACAGGAGAACGCTGGTAATCGGTTAGAAGAGCCTTCTTCCAACGTGACAGCAACGATGACGGTGTTACCTGGTGTGGTGACTGTGAAG |
| Insulin like growth factor binding protein 5 | *igfbp5* | TCTAATTCAAAGCATGAAGGACACTTCCAGGGTCTTGGCTCTCACTCTGCACATTCCCAACTGTGACAAGAAGGGCTTCTTCAAGCGCAAACAGTGTAAG |
| Luteinizing hormone receptor | *lhr* | CCTCATCACTGTCACCAACTCCAAGATTCTGCTGGTGCTCTTCTTCCCAATCAATTCCTGTGCCAATCCCTTCCTCTACGCTATCTTCACCAAGGCTTTC |
| Nanos | *nanos* | ATCTGGCTACTAAATCTACGCTGCCAAACCCTTTGGCTCATCAGCGTCCACCTGATGGTTTGCGGTACGCTTCAGATTCCCTAGGTGCTAATACACCAGA |
| Nuclear receptor subfamily 0 group B member 1 | *nrs0b1* | GCATATCTGAAAGGAGCTGTGCTGTTCAACCCAGATGTGGAAGGTTTGCGCTGTCTTCACTACATCCAGTCTCTGCGTCGGGAGGCGCACCAGGCTTTAA |
| P43 5S RNA-binding protein | *42sp43* | CAAAAAGCGCAGAATGTTTAAACTGCACTTACAGGAGCATGAAGTGGCTGCTAAATTCAAGTGTCTGAAGGACGGATGCGCTGCCACGTTCGACTCCCAT |
| Relaxin receptor 2 | *rr2* | TAAGAGCAGAGAACAACCTGCATGCTGCCTGCATCAAAGTCCTCTGTTGTGCAGACTGCCTCATGGGTGTGTACCTGTTTTTTGTTGGAGTGTTCGATGT |
| Sperm flagellar protein 1 | *spef1* | GCTCCATTTGCGGTCCAGGCAGAGGTGACCCTCAAGACAAACTTCTTCGCCACCAGAGACATGTTGACTCACTTCCTGCCGATCATCAAAGCTGGAGGCC |
| Sperm flagellar protein 2 | *spef2* | CACTATCTTGCAGAAATGAAGAGTATTGACCAACTATCAGAGGTGGTTCGTCACCACATAGAGGCTGGTGCTAAGTTGCAGAATCAGCTGTTATTGGAAT |
| Sperm surface protein SP17 | *sp17* | GCAGCTAATGAAAAAGACAGCATCTCTGTTCCAGACCAAGATATACCTCAGTCTAAGTTAGAGCCCACAGACTTATTATCATACAGAGGGGTTTCAGGTA |
| SRY-box transcription factor 7 | *sox7* | GGGGGCACACATATAGGTCACATCCCTCACATGTCCCAAACTGGAAGTGGCGGTGGACTGATCCCTGGCCATCCGCTGTCTTACTACAGTACCTCATCTT |
| SRY-box transcription factor 9B | *srybox9b* | CTGGCCGATCAATACCCGCATCTGCACAACGCGGAACTCAGCAAAACCCTGGGCAAACTTTGGAGATTGCTCAACGAAGTAGAGAAGCGCCCGTTTGTGG |
| Steroidogenic Acute Regulatory Protein | *star* | AGATGTTGCCTGACATTGGGAAGGTGTTCAAGCTGGAAGTGATGTTGGAGCAGCGTCCTGACAATCTTTACAAAGAGCTGGTGGGAAATATGGAGCAAAT |
| Synaptonemal complex protein 1 | *sycp1* | ACCTTTCAGACGGAGCAACTAAGGAAGGACATCACACAGCATGAAGTAAAGTACAAAGAACTATTATCCAACTTTAACGAGCTCCAGTCTGAGAAGGCAG |
| Uncharacterized protein 1 | *up1* | ATGAACCCAGCTACGCCTCCAGTGAGCAGATTACCAGCAGTACAGGCTCTCTCCAAGCCTCTTGGAACAGCAGGAACTCTGCCCAGGGAGCTCGTAACCT |
| Uncharacterized protein 2 | *up2* | CTGGACATCAACTACACACTGACTGTTCTGCCTGAAAACGAACTGTATTACCACCTGGCATCTATTGAGGCATTGTTCACAGATGTCTATCCTCCATCCT |
| Vitellogenin | *vtg* | AAATACTTCAAGTCGTATGACAATGGTGACTCTCAGCTGGACTCCTCAGAGCTGCTCAAATTCATCCAGCAAAATGACTCGGTTGTGGAGTTGCAGTCTT |
| Vitellogenin C | *vtgc* | TTCCAAGAAAGCCACCCAGCGAGTACGTGTGTCCTCTGATTCAGCCTCAAGCGTCAGAGAGAGATCTAATCACAGCCATCATGACAATATAATGGAAAGC |
| Wnt family member 5B | *wnt5b* | GGGCGGTTGCGGCGACAACGTACATTATGGCTACCGATTTGCCCGGGAGTTTGTGGACGCCAGGGAGAGAGAGAAGAATTACCCGCGTGGTTCTGCTGAG |
| Zona pellucida 3 | *zp3* | TGGGAGTCATCTGTTGTACACCAATGATCTGACCTACATTTCCTCTCCCGATTCTAACATTCTCCCGTCCACTCACCTAGTTGTCTGTGAGTATGAGAGG |
| Zona pellucida 3iX1 | *zp3ix1* | CAAGAGTTATGCTGTGGATGGCATTTCCCTGCATCCCACCTGGGTTCCTTTTGTCAAAACAGCCTCAGCAGAGGATCAGCTGGTCTTCAATTTGCGAACC |
| Zona pellucida 4 | *zp4* | GACACTGAAGCTCTACAAGTCGTATTTGGATCTCTGTACCGGGATGATGTTCTGATCAAGCCCAGCAGAGTCGTCAGTATTCTTGCCGCTGCTTGTATGC |
| Zona pellucida AX | *zpax* | GGTTACAGTCTACTCCCTCCCTCTGATCTTTGGGCTCATTATCCTACCTGAACAAATGCAGTTCACTCACTCAGTGGATTTGCAGGCGTCTCTGCAGGAT |
| Zygote arrest 1-like | *zar1* | GGGTCCAACTTTCAGTTCCTAGAGCAGAGGTATGGCTTTTTCCACTGCAAAAAGTGTAACATCCGGTGGGAGAGTGCTTATGTGTGGTGCATCTCTGGAA |
