# Supplementary material for: A case study: temporal trends of environmental stressors and reproductive health of smallmouth bass (Micropterus dolomieu) from a site in the Potomac River Watershed, Maryland, USA
Source: Ecotoxicology. 2022 Dec 1;31(10):1536–53. doi: 10.1007/s10646-022-02605-8 (PMC9729326; doi:10.1007/s10646-022-02605-8)
Supplement: Supplementary file 4 — Supplementary Table 3 [file 10646_2022_2605_MOESM4_ESM.docx]

A Case Study: Temporal Trends of Environmental Stressors and Reproductive Health of Smallmouth Bass (*Micropterus dolomieu*) from a Site in the Potomac River Watershed, MD, USA

Heather L. Walsh*^1^, Stephanie E. Gordon^1^, Adam J. Sperry^1^, Michael Kashiwagi^2^, John Mullican^3^, and Vicki S. Blazer^1^

^1^U.S. Geological Survey, Eastern Ecological Science Center, Leetown Research Laboratory, 11649 Leetown Rd., Kearneysville, West Virginia 25430, USA

Corresponding author: *hwalsh@usgs.gov

^2^Maryland Department of Natural Resources, Fishing and Boating Services, 10932 Putman Rd., Thurmont, Maryland 21788, USA

^3^ Maryland Department of Natural Resources, Fishing and Boating Services, 20901 Fish Hatchery Rd., Hagerstown, Maryland 21740, USA

ORCID ID:

Heather L. Walsh 0000-0001-6392-4604

Stephanie E. Gordon 0000-0002-6292-2612

Adam J. Sperry 0000-0002-4815-3730

Vicki S. Blazer 0000-0001-6647-9614

**KEYWORDS**

Reproductive endocrine disruption, testicular oocytes, plasma vitellogenin, contaminants, land use, long-term monitoring

**ACKNOWLEDGEMENTS**

We would like to thank the electroshocking crew from Area 7 of the Western Region of the Maryland Department of Natural Resources for assistance in the collection of smallmouth bass used in this study. We also thank the graduate students and technicians who have helped provide field, histology, and laboratory assistance. Any use of trade, product, or firm names is for descriptive purposes only and does not imply endorsement by the U.S. Government.

| Date | *3βhd* | *42sp43* | *up1* | *cypp45011b* | *dmrt2* | *dmrt3* | *sox11* | *star* | *up2* | *zp3* | *zp4* | *zpax* | *arα* | *erα* | *inhα* | *a-fabp* | *socs3* | *tgfβ2* | *tollip* |
| --- | --- | --- | --- | --- | --- | --- | --- | --- | --- | --- | --- | --- | --- | --- | --- | --- | --- | --- | --- |
| Spring 2015 | 2300 | 101 | 130 | 224 | 383 | 114 | 100 | 449 | 245 | 246 | 123 | 162 | 101 | 55 | 5618 | 321 | 326 | 473 | 1551 |
| Spring 2016 | 2603 | 315 | 525 | 639 | 1136 | 457 | 347 | 1171 | 1623 | 957 | 395 | 639 | 315 | 53 | 6599 | 854 | 691 | 1055 | 921 |
| Fall 2016 | 1653 | 830 | 719 | 458 | 487 | 263 | 295 | 773 | 1185 | 1116 | 325 | 588 | 830 | 68 | 6158 | 175 | 825 | 213 | 204 |
| Spring 2017 | 2171 | 902 | 616 | 665 | 704 | 369 | 291 | 931 | 1144 | 971 | 321 | 554 | 902 | 79 | 8664 | 460 | 841 | 332 | 428 |
| Fall 2018 | 1887 | 557 | 253 | 405 | 612 | 205 | 167 | 381 | 595 | 434 | 206 | 239 | 557 | 92 | 8564 | 977 | 803 | 540 | 2351 |

|  | Plasma Vtg | *17βhd* | *arα* | *arβ* | *chg* | *erα* | *erβ1* | *erβ2* | *vtg* |
| --- | --- | --- | --- | --- | --- | --- | --- | --- | --- |
| Spring 2013 | 124.05 | 124 | 46 | 235 | 237 | 82 | 79 | 218 | 178 |
| Spring 2015 | 86.10 | 113 | 34 | 434 | 2046 | 170 | 52 | 324 | 182 |
| Spring 2016 | 41.35 | 104 | 41 | 277 | 125 | 110 | 55 | 254 | 129 |
| Spring 2017 | 27.99 | 178 | 78 | 301 | 322 | 100 | 88 | 208 | 96 |
| Spring 2018 | 10.98 | 125 | 37 | 358 | 788 | 122 | 42 | 272 | 221 |
| Spring 2019 | 41.06 | 132 | 57 | 161 | 904 | 181 | 67 | 106 | 60 |
| Fall 2016 | 23.03 | 188 | 36 | 204 | 289 | 192 | 58 | 249 | 100 |
| Fall 2018 | 13.59 | 104 | 40 | 161 | 310 | 79 | 45 | 190 | 67 |
|  |  |  |  |  |  |  |  |  |  |
|  | Plasma Vtg | *17βhd* | *arα* | *arβ* | *chg* | *erα* | *erβ1* | *erβ2* | *vtg* |
| Spring 2013 | 2068 | 362 | 164 | 146 | 80940 | 848 | 116 | 225 | 422784 |
| Spring 2015 | 2044 | 433 | 149 | 267 | 183277 | 891 | 155 | 519 | 767055 |
| Spring 2016 | 862 | 450 | 144 | 71 | 170803 | 1281 | 124 | 435 | 563426 |
| Spring 2017 | 249 | 148 | 81 | 413 | 53797 | 472 | 102 | 232 | 257149 |
| Spring 2018 | 172 | 399 | 127 | 141 | 147440 | 1044 | 101 | 397 | 474609 |
| Spring 2019 | 318 | 186 | 102 | 213 | 125964 | 876 | 140 | 130 | 503647 |
| Fall 2016 | 145 | 167 | 49 | 224 | 23656 | 367 | 48 | 256 | 113863 |
| Fall 2018 | 132 | 178 | 52 | 136 | 39603 | 437 | 50 | 198 | 150700 |
